# Neuronal autoantibodies associated with poorer neuropsychological and motor outcomes 6 months after stroke: results from the PHYS-STROKE trial

**DOI:** 10.3389/fimmu.2025.1678840

**Published:** 2026-03-18

**Authors:** Charlotte Pietrock, Konrad Neumann, Kristin Rentzsch, Harald Prüss, Andreas Meisel, Matthias Endres, Alexander Heinrich Nave

**Affiliations:** 1 Department of Neurology with Experimental Neurology, Charité – Universitätsmedizin Berlin Corporate Member of Freie Universität Berlin, Humboldt-Universität zu, and Berlin Institute of Health, Berlin, Germany; 2 Center for Stroke Research Berlin, Charité – Universitätsmedizin Berlin, Berlin, Germany; 3 Institute of Biometry and Clinical Epidemiology, Charité – Universitätsmedizin Berlin, Berlin, Germany; 4 Clinical Immunological Laboratory Prof. h.c. (RCH) Dr. med. Winfried Stöcker, Groß Grönau, Germany; 5 German Center for Neurodegenerative Diseases (DZNE), Berlin, Germany; 6 Neuroscience Clinical Research Center, Charité – Universitätsmedizin Berlin, Berlin, Germany; 7 German Centre for Cardiovascular Research (DZHK), Berlin, Germany; 8 German Center for Mental Health (DZPG), Berlin, Germany; 9 Berlin Institute of Health, Berlin, Germany

**Keywords:** biomarker, neuroimmunology, neuronal autoantibodies, NMDAR, outcome, recovery, stroke

## Abstract

**Introduction:**

Emerging evidence suggests a role of neuronal autoantibodies (nAbs) for long-term stroke outcomes. However, data remain limited and many domains unexamined. We present a comprehensive analysis of nAbs and their association with a broad range of outcome measures at multiple timepoints in the six months following moderate stroke.

**Methods:**

In this explorative analysis of the multicenter, randomized-controlled *PHYS-STROKE* trial, serum samples from stroke patients were tested for 40 nAbs at baseline (5–45 days post-stroke), post-intervention (4 weeks after baseline), and at three and six months after stroke. Generalized estimating equation (GEE)-models were used to evaluate the dynamics of nAbs over time. Multiple linear regression models were applied to investigate the prognostic role of nAbs on various outcomes at three and six months.

**Results:**

Two hundred stroke patients (41% female; mean age: 69 ± 12 years, median acute National Institutes of Health Stroke Scale: 8) were enrolled. Cell-based seroreactivity decreased from baseline to six months (39 of 183 patients [21%] *vs.* 18 of 137 patients [13%]). while tissue-based reactivity increased (4 of 183 patients [2%] *vs.* 9 of 137 patients [7%]). The GEE applied to the imputed dataset indicated a statistically significant decreased likelihood of seroreactive nAbs in cell-based assays from baseline to six months (*95%CI* = 0.36 to 0.98; *p* = 0.041), while tissue-based analyses showed an inverse effect for the same time period (*95%CI* = 1.11 to 8.51; *p* = 0.032). The most frequently detected antibody was anti-N-Methyl-D-Aspartate receptor GluN1 (NMDAR (IgM, IgA, IgG), 30 patients [15.1%]). Baseline nAB seropositivity was associated with worse depression scores (*95%CI* = 0.03 to 7.82; *p* = 0.048) and poorer subjective mobility (*95%CI=*0.04 to 0.99; *p* = 0.033) at six months post-stroke. NMDAR-antibodies at baseline were linked to a lower subjective overall health rating (*95%CI* = -17.96 to -0.16; *p* = 0.046) and lower maximum walking speed (*95%CI* = -0.57 to -0.03; *p* = 0.027) at six months. No associations were found with outcomes at three months.

**Conclusions:**

Antibody seropositivity was associated with poorer outcomes in certain neuropsychological and motor outcome measures at six but not three months post-stroke. These findings require confirmation in larger cohorts and emphasize the need for future studies with longer follow-up periods in this patient population.

**Trial registration:**

clinicaltrials.gov NCT01953549.

## 1 Introduction

Stroke is the third leading cause of death, and fourth leading cause of disability-adjusted life-years worldwide (1). Residual deficits are common and include impaired language, motor and sensory function. Additionally, less overt but equally prevalent and clinically important symptoms— such as cognitive impairment, depression, anxiety, pain, sleep disorders and fatigue— further reduce autonomy, social functioning and quality of life. Due to population growth and ageing, combined with advances in acute stroke treatment resulting in increased survival rates, the number of people living with stroke and its long-term sequelae is expected to grow (1, 2). To mitigate the long-term burden of stroke, it is essential to identify factors that influence recovery and outcomes.

Neuronal autoantibodies are antibodies targeted against neuronal or glial cell proteins and have been found in both healthy and ill individuals (3–5). In stroke, knowledge on their clinical significance is limited and most studies have focused exclusively on the most common anti-N-Methyl-D-Aspartate receptor GluN1 (NMDAR) antibody (6). A previous study identified a dual role of NMDAR antibodies in stroke patients, with either beneficial or detrimental effects on lesion size evolution, depending on the blood-brain barrier integrity of patients, as indicated by APOE4 carrier status (7). A joint analysis of three independent prospective studies found that NMDAR antibody seroprevalence had no significant effect on functional outcome at 30 or 90 days after stroke (8). Sperber et al. reported a worse functional outcome one year after first-ever ischemic stroke in patients with high serum titers (>1:100) of NMDAR antibodies at stroke onset (9). Furthermore, patients with NMDAR antibodies showed an increased risk for secondary vascular events or death within the first three years following stroke (9). In addition to functional outcome, NMDAR seropositivity has been implicated in cognitive impairment across multiple diseases (3, 10–13). In stroke patients, the presence of NMDAR antibodies in the acute phase has been linked to long-term cognitive decline (14, 15). Recently, NMDAR seropositivity was found to be associated with increased risk for memory deficits 12 months after stroke (16). Additionally, higher rates of fatigue and depressive symptoms have been described in ischemic stroke patients with NMDAR antibodies (15, 17). In most of these studies, antibodies were measured once in the acute phase of stroke and were thus presumably pre-existing at the time of the index event. Data on antibody seroconversion or their prevalence in the subacute and chronic phase of stroke remain scarce (8, 18). Moreover, many studies predominantly included patients with mild to moderate stroke severity, limiting the ability to comprehensively assess long-term neurological and motor outcomes. To our knowledge, the impact of neuronal autoantibodies on long-term quality of life after stroke has not been investigated.

Here, we conducted a thorough antibody profiling in serial measurements up to six months following stroke. Our aim was to assess the dynamics of neuronal autoantibodies over time and explore their association with a broad range of motor and non-motor outcome measures.

## 2 Materials and methods

### 2.1 Patient population

This exploratory analysis utilized data from the “Physical Fitness Training in Patients with Subacute Stroke” (*PHYS-STROKE*) trial (clinicaltrials.gov identifier: NCT01953549) (19, 20). In this multicenter, randomized-controlled, endpoint-blinded clinical trial, 200 subacute stroke patients were enrolled at seven in-patient rehabilitation centers in Berlin and Brandenburg, Germany, between September 2013 and April 2017. Adult patients with imaging-confirmed ischemic or hemorrhagic stroke, a baseline Barthel Index (BI) ≤ 65 and deemed ability to comply with the study requirements were included in the study. The complete inclusion and exclusion criteria are available in the **Supplementary Materials, Table S1**. All patients provided written informed consent. The study was conducted in accordance with the Declaration of Helsinki and was approved by the local ethics review board of the Charité – Universitätsmedizin Berlin (EA1/138/13). The study adhered to “Strengthening the Reporting of Observational Studies in Epidemiology” (STROBE) guidelines for research reporting (21).

### 2.2 Study design and outcome measures

The *PHYS-STROKE* trial compared four weeks of physical fitness training to additional relaxation treatment in addition to standard in-patient rehabilitation treatment. Patients were enrolled within 5–45 days after stroke onset and received clinical visits pre-intervention (baseline - BL), as well as post-intervention (visit 1 - V1), three (visit 2 - V2) and six (visit 3 - V3) months after stroke. The intervention comprised either four weeks of bodyweight-supported, treadmill-based aerobic exercise for 25 minutes, five days a week or muscle relaxation exercises of the same duration and frequency. Various outcome parameters were collected at each visit (19, 20). The co-primary outcome measures were the change in BI and maximal walking speed from BL to V2. For this exploratory analysis, we examined all primary and secondary outcome measures collected in the *PHYS-STROKE* trial. Sample characteristics (**Table S2**) are detailed in the **Supplementary Materials**. A subsample of 110 patients additionally participated in the “Biomarkers and Perfusion – Training-Induced Changes After Stroke” (*BAPTISe*) trial (clinicaltrials.gov identifier: NCT01954797) which entailed additional blood- and MRI-measurements pre- and post-intervention (22). The complete study design and main results of both studies are detailed in prior publications (19, 20, 22).

### 2.3 Neuronal autoantibody analysis

Serum samples were collected and aliquoted at each clinical visit. The samples were frozen at -80 °C until they were thawed for autoantibody measurements. Neuronal autoantibodies were identified via indirect immunofluorescence using standardized commercial assays (BIOCHIP mosaicsTM, EUROIMMUN) at the Clinical Immunological Laboratory Prof. h.c. (RCH) Dr. med. Winfried Stöcker, Groß Grönau, Germany. The assays contained recombinant HEK293-cells each expressing one of 40 specific neuronal antigens (see **Table S3**, “cell-based assays”) or frozen tissue of monkey and rat cerebellum (“tissue-based assays”). Patient serum was serially diluted (at 1:2, 1:10, 1:32, 1:100, 1:320, 1:1000, 1:3200) and applied to the cell- and tissue-based assays, followed by incubation with fluorescein isothiocyanate antihuman immunoglobulin. For NMDAR detection, anti-IgM, anti-IgA and anti-IgG isotypes were used, while anti-IgG alone was applied for all other antigens. The immunofluorescence patterns were interpreted, and microscopic analysis performed by an investigator blinded to patient data within 24 hours of incubation. To validate reactivity for all intracellular antigens, immunoblot assays were additionally performed. Titers ≥ 1:10 were considered seropositive. Titers between ≥1:10 and <1:100 were defined as *low titers* and titers ≥ 1:100 as *high titers*.

### 2.4 Statistical analysis

All statistical analyses were conducted using the R software environment (version 4.1.2, R Core Team) (23). The significance-level was set at α = 0.05. Pearson’s Chi-square test was applied to categorical data, while the Mann-Whitney test was used for quantitative data. To assess changes in serostatus prevalence over a 6-month period and its association with the intervention (physical fitness training *vs.* relaxation treatment) as well as patient- and stroke-specific factors (e.g. age, sex, National Institutes of Health Stroke Scale (NIHSS), history of cerebrovascular disease (stroke and/or transient ischemic attack) generalized estimating equation (GEE) models were applied. Age was categorized into three groups (≤ 65 years, 66–75 years, ≥ 76 years) prior to analysis, due to two outlier observations (patients < 40 years of age). Multivariate linear regression models were used to evaluate whether baseline serostatus predicted outcome measures at 3 and 6 months post-stroke. Possible confounders included age, baseline stroke severity as assessed by the NIHSS, corresponding baseline test scores and educations level where applicable. The intervention was not the primary variable of interest in this exploratory study and excluded as a confounder in the linear regression models to reduce overfitting. Missing values in the regression and GEE models were accounted for using multiple imputation with the R-package “*mice*”, based on 1000 imputed datasets and 5 iterations. To account for multiple comparisons, *p*-values of the imputed models were additionally adjusted using the Benjamini-Hochberg procedure.

## 3 Results

### 3.1 Patient population

A total of 200 stroke patients (female: *n* = 81 [41%]; age: *M*(SD) = 69 (12) years) were enrolled in the *PHYS-STROKE* trial. Ischemic strokes (*n* = 181 [91%]) were more prevalent than hemorrhagic strokes. The majority of patients (*n* = 162 [81%]) had a first-ever stroke. The stroke severity of the cohort was moderate, with a median baseline NIHSS of 8 (Q1 25% – Q3 75%: 5 – 12). Accordingly, the degree of disability at enrollment was high, with a median mRS of 4 (4 – 4) and a median BI of 50 (35 – 60). One hundred five patients [53%] were randomized to the aerobic fitness group, and 95 patients [47%] to the relaxation group. No statistically significant differences were observed between the aerobic fitness and relaxation group regarding their impact on maximal walking speed and activities of daily living three months after stroke (19). The aerobic fitness group had a higher incidence of serious adverse events compared to the relaxation group at three months after stroke (19, 24).

### 3.2 Antibody frequency and titer development

**Figure 1:**
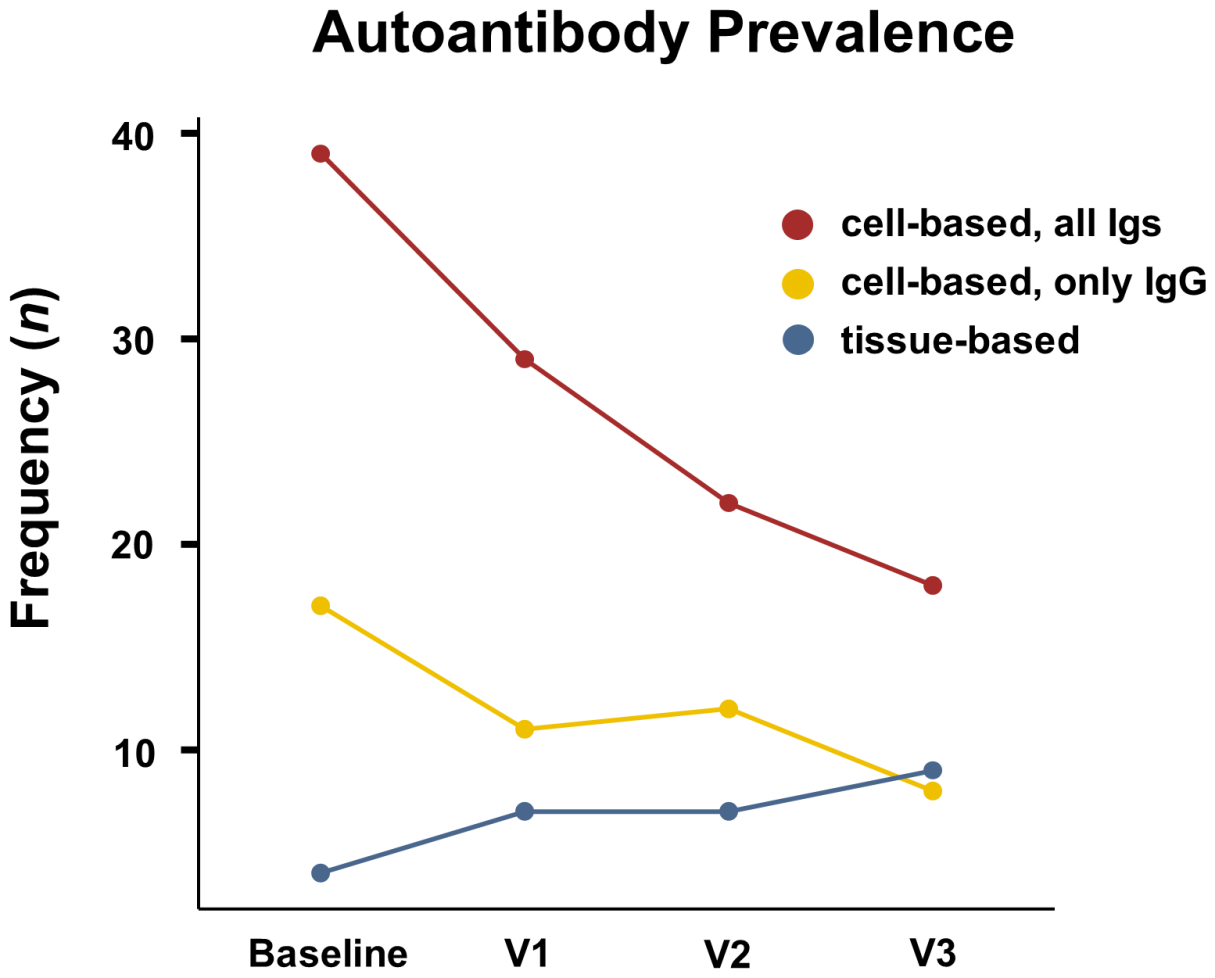
Autoantibody prevalence. Development of autoantibody prevalence in cell-based analyses for all isotypes (red), cell-based analyses regarding only IgG isotypes (yellow) and tissue-based analyses (blue) over the course of 6 months after stroke. Timepoints are pre-intervention (baseline), post-intervention (V1), three (V2) and six (V3) months after stroke.

One hundred ninety-nine patients received antibody analyses at one or more timepoints and 109 patients [55%] had measurements at all four timepoints. One hundred eighty-three patients [92%] received antibody analyses at baseline at a median of 25 days after stroke (15–37 days). Only a minority of patients (*n* = 44) received antibody testing in the first two weeks after stroke. The number of patients with BL, V1, V2 and V3 blood analyses, as well as the respective median days after stroke are detailed in **Table 1**. The underlying study population in this table is not identical at each timepoint, as the follow-up rate decreased with time. In summary, 39 patients [21%] showed seroreactivity in cell-based assays at baseline for all isotypes and this number decreased to 18 patients [13%] by six months after stroke (V3). A similar development was observed when examining only patients with high-titer antibodies or only IgG isotypes. In contrast, reactivity in tissue-based analyses was low at baseline (*n* = 4 patients [2%]) and increased over time (*n* = 9 patients [7%] by V3). This divergent course in frequency of reactivity in cell-based- and tissue-based-analyses is depicted in **Figure 1**. An examination including only patients (*n* = 109) that received complete antibody analyses at all visits showed comparable results (**Table S4**). A list of all autoantibody types detected in the cohort can be found in **Table 1**. The median time of baseline blood sampling was 25 days (15.8 – 36.3) and did not statistically differ (*p* = 0.290) between seropositive (21 days, 13-5 – 35.5) and seronegative (26 days (16.8 – 37.0) patients.

**Table 1:** Neuronal autoantibody frequencies.

	Any timepoint (*n* = 199)	Baseline (*n* = 183)	V1 (*n* = 162)	V2 (*n* = 150)	V3 (*n* = 137)
Time after stroke (days, median, Q1 25% - Q3 75%)	-	25 (15 – 37)	57 (44 – 70)*	91 (87 – 94)	180 (177 – 184)
Tissue-based reactivity summarized (*n*, %)	14 (7.0)	4 (2.2)	7 (4.3)	7 (4.7)	9 (6.6)
Cell-based reactivity summarized (all Igs, *n*, %)	48 (24.1)	39 (21.3)	29 (17.9)	22 (14.7)	18 (13.1)
Cell-based seroreactivity summarized (only IgG, *n*, %)	22 (11.1)	17 (9.3)	11 (6.8)	12 (8.0)	8 (5.8)
High-titer (≥1:100) cell-based seroreactivity summarized (all Igs, *n*, %)	26 (13.1)	17 (9.3)	15 (9.3)	10 (6.7)	7 (5.1)
High-titer (≥1:100) cell-based seroreactivity summarized (only IgG, *n*, %)	12 (6.0)	8 (4.4)	8 (4.9)	4 (2.7)	2 (1.5)
NMDAR Igs summarized (*n*, %)	30 (15.1)	24 (13.1)	19 (11.7)	12 (8.0)	12 (8.8)
NMDAR IgM (*n*, %)	21 (10.6)	16 (8.7)	14 (8.6)	10 (6.7)	6 (4.4)
NMDAR IgA (*n*, %)	14 (7.0)	10 (5.5)	9 (5.6)	7 (4.7)	8 (5.8)
NMDAR IgG (*n*, %)	2 (1.0)	2 (1.1)	0	1 (0.7)	1 (0.7)
NMDAR high-titer (≥1:100), all Igs summarized (*n*, %)	14 (7.0)	9 (4.9)	7 (4.3)	6 (4.0)	5 (3.6)
Homer3 IgG (*n*, %)	4 (2.0)	3 (1.6)	2 (1.2)	1 (0.7)	0
GLRA1b IgG (*n*, %)	4 (2.0)	3 (1.6)	3 (1.9)	3 (2.0)	2 (1.5)
Flotillin IgG (*n*, %)	3 (1.5)	2 (1.1)	2 (1.2)	2 (1.3)	1 (0.7)
CASPR2 IgG (*n*, %)	3 (1.5)	2 (1.1)	0	1 (0.7)	1 (0.7)
KCNA2 IgG (*n*, %)	1 (0.5)	1 (0.5)	1 (0.6)	1 (0.7)	1 (0.7)
Neurochondrin IgG (*n*, %)	1 (0.5)	1 (0.5)	1 (0.6)	1 (0.7)	0
MOG IgG (*n*, %)	1 (0.5)	1 (0.5)	1 (0.6)	1 (0.7)	1 (0.7)
AP3B2 IgG (*n*, %)	1 (0.5)	1 (0.5)	0	1 (0.7)	1 (0.7)
ITPR1 IgG (*n*, %)	1 (0.5)	0	1 (0.6)	0	0
GRM5 IgG (*n*, %)	1 (0.5)	0	0	0	1 (0.7)
DRD2 IgG (*n*, %)	1 (0.5)	1 (0.5)	0	0	0

No reactivity found for: GAD65 IgG, GABA-b IgG, Aquaporin 4 IgG, LGI1 IgG, GluRD2 IgG, GABA-a IgG, AMPAR IgG, GRM1 IgG, Ma2 IgG, Recoverin IgG, Zic-4 IgG, Iglon 5 IgG, DPPX IgG, AT1A3 IgG, Neurofascin 155 IgG, Neurofascin 186 IgG, CNTN1 IgG, CV2 IgG, Hu IgG, Ri IgG, ERC1 IgG, Rho-GTPase 26 IgG, DNER IgG, Sez6I2 IgG, CARPVIII IgG, Amphiphysin IgG, Yo IgG, Neurexin 3-alpha IgG. * one date missing.

#### 3.2.1 Anti-NMDAR autoantibodies

The most common autoantibody observed in cell-based assays was NMDAR (*n* = 30 patients [15.1%]). NMDAR-IgM isotypes (*n* = 21 [10.6%]) were more prevalent than NMDAR-IgA (*n* = 14 [7.0%]) and NMDAR-IgG (*n* = 2 [1.0%]). The incidence of NMDAR-IgM autoantibodies decreased over time; this observation was less pronounced for NMDAR-IgA isotypes. The titer development of NMDAR-IgM and -IgA over time on a logarithmic scale can be found in the **Supplementary Materials** (**Figure S1**). A detailed characterization of the two NMDAR-IgG seroreactive patients can be found in **Table S5**. Five patients [2.5%] were positive for NMDAR-IgM and NMDAR-IgA antibodies, while one patient [0.5%] showed reactivity for all three NMDAR isotypes.

**Figure 2:**
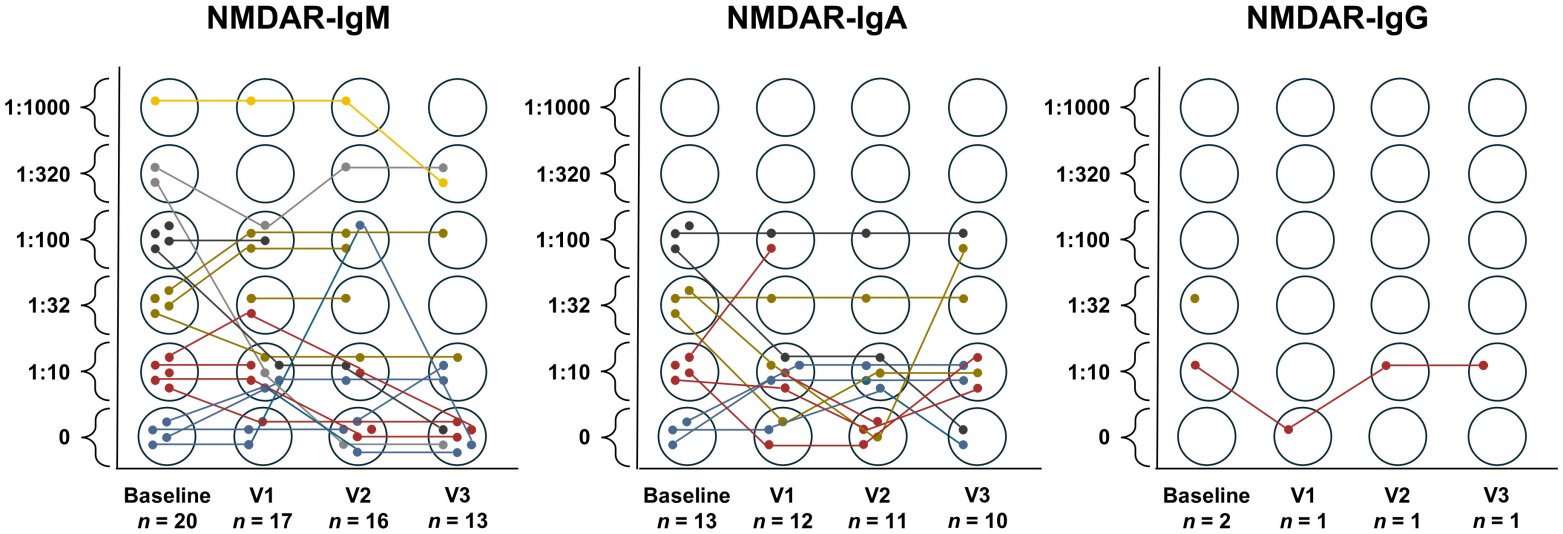
Development of titer over time by NMDAR-isotype. The colours correspond to the titer at baseline to simplify tracking the individual titer progression.

The individual course of titer development by NMDAR isotype is displayed in **Figure 2**. In the group showing NMDAR-IgM seroreactivity (*n* = 21 [10.6%]), 4 patients [19.0%] had consistently detectable NMDAR-IgM antibodies at all timepoints. Five [23.8%] initially NMDAR-IgM seropositive patients lost seroreactivity during follow-up. In contrast, only 2 patients [9.5%] seroconverted with time, both to a titer level of 1:10. There were 2 patients [9.5%] who began and terminated the study seronegative but had intermittently detectable NMDAR-IgM antibodies to a titer level of 1:10 and 1:100. No concrete conclusion can be drawn about 8 patients [38.1%] as they did not have antibody measurements at all timepoints.

NMDAR-IgA antibodies were consistently detectable at all four timepoints in 2 [14.3%] of 14 NMDAR-IgA seropositive patients in total. The same number of patients seroconverted, again to a titer level of 1:10. One patient [7.1%] lost NMDAR-IgA seroreactivity during follow-up. Another patient [7.1%] had only intermittently detectable NMDAR-IgA antibodies at a titer level of 1:10. Four patients [28.6%] began and terminated the study with NMDAR-IgA antibodies but had one or two measurements in between where no antibodies were detected. Four patients [28.6%] had incomplete antibody measurements.

#### 3.2.2 Other neuronal autoantibodies

Aside from NMDAR autoantibodies, seroreactivity in cell-based assays was found for 11 further neuronal autoantibodies in 20 patients [10.1%] over all timepoints (**Table 1**). These patients are characterized in detail in the **Supplementary Materials** (**Table S5**). There was no discernable common clinical characteristic among these patients. Three patients [1.5%] were seroreactive for more than one autoantibody type (NMDAR-IgA and CASPR2; NMDAR-IgM and Homer; GRM5 and GLRA1b).

#### 3.2.3 Tissue-based analyses

Reactivity in tissue-based analyses was found in 14 patients [7.0%]. Three [21.4%] of these patients showed reactivity at all visits, 5 [35.7%] at two timepoints, and 6 [42.9%] at one timepoint. Of the patients with tissue-based assay reactivity, 4 patients [28.6%] exhibited specific antibody patterns (2 GFAP, 1 KCNA, 1 Flotillin). The remaining 10 patients [71.4%] showed unspecific staining patterns. Among these, 4 patients [40.0%] showed reactivity against Myelin structures, another 4 patients [40.0%] showed staining of the finely granular molecular layer and/or patchy granular layer, and 3 patients [30.0%] showed a pattern involving Purkinje neurons. Three [21.4%] of the 14 patients with reactivity in tissue-based analyses also showed reactivity in cell-based analyses.

### 3.3 Baseline characteristics

The baseline characteristics of patients seroreactive for the most common neuronal autoantibody (NMDAR) in cell-based assays were compared to patients with no observable autoimmune activity at baseline (**Table S6**). Patients with NMDAR autoantibodies (all isotypes) at baseline were statistically significantly more likely to have a history of cerebrovascular disease (50% *vs.* 26%; *p* = 0.036), especially transient ischemic attacks (29% *vs.* 10%; *p* = 0.028). NMDAR-seropositive patients showed statistically significantly higher serum triglyceride levels at baseline compared to seronegative patients (1.7 ± 0.5 mmol/l *vs.* 1.4 ± 0.6 mmol/l; *p* = 0.002). Additionally, a trend towards lower serum HDL-cholesterol levels at baseline was also observed in the NMDAR-seropositive group (1.1 ± 0.4 mmol/l *vs.* 1.3 ± 0.3 mmol/l; *p* = 0.064). NMDAR-seropositive patients showed statistically significantly lower mRS scores at baseline compared to seronegative patients (4 (3 – 4) *vs.* 4 (4 – 4); p = 0.011). There was a trend towards a higher proportion of females in the NMDAR-seropositive group (58% *vs.* 38%; *p* = 0.089). No other statistically significant differences regarding patient demographics, stroke characteristics (including normalized stroke lesion volume compared in non-parametric testing in the *BAPTISe* subsample) or cardiovascular risk factors were observed between the two groups. There were no statistically significant differences between the two groups regarding the occurrence of serious adverse events (hospital referral, serious cerebrovascular event, serious cardiovascular event, death) over the course of the study (**Table S7**).

### 3.4 Factors influencing neuronal autoantibody prevalence over time

The results of the original and imputed GEE-models are displayed in **Table 2**. Compared to baseline, the likelihood of any seroreactive neuronal autoantibodies in cell-based assays was statistically significantly decreased by 6 months after stroke (*95% CI* = 0.36 to 0.98; *p* = 0.041). GEE-models of tissue-based analyses showed an inverse effect, with a statistically significantly increased likelihood of seroreactivity 6 months after stroke compared to baseline (*95% CI* = 1.11 to 8.51; *p* = 0.032, **Table S8**). Considering only NMDAR-antibodies, female patients showed a three times higher odds (*95% CI* = 1.34 to 6.71; *p* = 0.008) of seroreactivity compared to male patients. Additionally, patients with a history of cerebrovascular disease were more likely to be NMDAR-seroreactive (*95% CI* = 1.04 to 5.43; *p* = 0.039). The study intervention (physical fitness training), NIHSS and age had no statistically significant effects on neuronal autoantibody seroreactivity.

**Table 2:** Factors associated with neuronal autoantibody seropositivity in cell-based analyses over time.

	Original model			Imputed model			
	OR	95% CI	*p*-value	OR	95% CI	*p*-value	Adjusted *p*-value
All antibodies							
Time (reference: baseline)							
Post-intervention (V1)	0.81	0.59 – 1.11	0.189	0.80	0.55 – 1.17	0.260	0.468
3 months post-stroke (V2)	0.65	0.43 – 0.97	**0.035**	0.66	0.42 – 1.05	0.077	0.284
6 months post-stroke (V3)	0.59	0.39 – 0.89	**0.012**	0.59	0.36 – 0.98	**0.041**	0.246
Age group (reference: ≤ 65 yrs)							
Age 66–75 yrs	2.16	0.87 – 5.33	0.097	2.09	0.92 – 4.72	0.079	0.284
Age ≥ 76 yrs	1.75	0.69 – 4.42	0.237	1.72	0.75 – 3.95	0.202	0.456
Sex (reference: male)	1.27	0.63 – 2.58	0.508	1.25	0.67 – 2.35	0.487	0.647
Intervention (reference: fitness)	1.57	0.79 – 3.13	0.197	1.54	0.84 – 2.82	0.165	0.424
NIHSS_baseline_	0.97	0.90 – 1.04	0.420	0.97	0.91 – 1.04	0.367	0.551
History of cerebrovascular disease (reference: negative)	1.28	0.59 – 2.79	0.538	1.25	0.63 – 2.47	0.530	0.647
NMDAR antibodies							
Time (reference: baseline)							
Post-intervention (V1)	0.90	0.61 – 1.33	0.597	0.88	0.56 – 1.39	0.575	0.647
3 months post-stroke (V2)	0.61	0.35 – 1.05	0.076	0.61	0.33 – 1.11	0.106	0.318
6 months post-stroke (V3)	0.69	0.42 – 1.15	0.156	0.70	0.38 – 1.29	0.248	0.468
Age group (reference: ≤ 65 yrs)							
Age 66–75 yrs	1.41	0.44 – 4.49	0.564	1.38	0.48 – 3.92	0.549	0.647
Age ≥ 76 yrs	1.14	0.36 – 3.57	0.824	1.15	0.41 – 3.21	0.789	0.789
Sex (reference: male)	3.08	1.24 – 7.66	**0.015**	3.00	1.34 – 6.71	**0.008**	0.144
Intervention (reference: fitness)	1.55	0.61 – 3.92	0.358	1.50	0.68 – 3.30	0.313	0.512
NIHSS_baseline_	0.98	0.89 – 1.09	0.730	0.99	0.90 – 1.08	0.737	0.780
History of cerebrovascular disease (reference: negative)	2.39	0.95 – 6.05	0.065	2.38	1.04 – 5.43	**0.039**	0.246

*OR* Odds Ratio; *95% CI* 95% Confidence Interval; *NIHSS* National Institutes of Health Stroke Scale.

Bold values indicate statistical significance (p < 0.05).

### 3.5 Association of neuronal autoantibodies with outcome

The original and imputed results from the multivariate linear regression models for outcome measures 6 months post-stroke are summarized in **Table 3**. Presence of neuronal autoantibodies at baseline was associated with a statistically significantly worse center for epidemiological studies depression scale (CES-D) score 6 months after stroke (*95% CI* = 0.03 to 7.82; *p* = 0.048). Similarly, in the EQ-5D-5L-score, seroreactive patients rated their mobility statistically significantly poorer compared to seronegative patients 6 months post-stroke (All antibodies: *95% CI* = 0.04 to 0.99; *p* = 0.033; only NMDAR antibodies; *95% CI* = 0.45 to 1.63; *p* = 0.001). A positive NMDAR serostatus was associated with a statistically significantly worse subjective overall health rating on the visual analogue scale of the EQ-5D-5L (95% CI = -17.96 to -0.16; *p* = 0.046) as well as a lower maximum walking speed at 6 months post-stroke (*95% CI* = -0.57 to -0.03; *p* = 0.027). After adjusting for multiple comparisons, only the poorer EQ-5D-5L mobility rating for patients with initial positive NMDAR serostatus remained statistically significant (*p* = 0.046). No further associations were observed between baseline serostatus and other outcomes measures of motor or non-motor domains at 6 months after stroke. The influence of NMDAR serostatus on mobility rating and overall subjective health was replicated in the crude models (**Table S9**). The crude models additionally showed a statistically significantly higher pain rating at 6 months after stroke in patients seroreactive for NMDAR at baseline (*95% CI* = 0.12 to 1.37; *p* = 0.019, **Table S9**). There was no statistically significant effect of antibody serostatus on outcome measures at three months after stroke (**Table S10**). In a subgroup analysis comparing the effect of antibody serostatus on outcome measures dependent on stroke type (ischemic *vs.* hemorrhagic) we discovered that the associations were all driven by patients with ischemic stroke (**Table S11**).

**Table 3:** Influence of neuronal autoantibodies (all or NMDAR only) on outcome measures at 6 months post-stroke.

	Original model			Imputed model			
	ß	95% CI	*p*-value	ß	95% CI	*p*-value	Adjusted *p*-value
mRS _All_	0.29	-0.12 – 0.69	0.162	0.22	-0.14 – 0.59	0.225	0.644
mRS _NMDAR_	0.14	-0.36 – 0.64	0.585	0.09	-0.37 – 0.54	0.714	0.853
BI _All_	-3.67	-10.35 – 3.02	0.280	-3.66	-10.57 – 3.26	0.297	0.646
BI _NMDAR_	-0.63	-9.25 – 8.00	0.886	-0.48	-9.28 – 8.33	0.915	0.963
CES-D _All_	4.71	0.79 – 8.62	**0.019**	3.93	0.03 – 7.82	**0.048**	0.407
CES-D _NMDAR_	5.78	0.94 – 10.62	**0.020**	4.12	-0.87 – 9.12	0.104	0.460
MoCA _All_	0.65	-0.73 – 2.03	0.351	0.83	-0.75 – 2.40	0.301	0.646
MoCA _NMDAR_	1.40	-0.32 – 3.11	0.110	1.60	-0.33 – 3.54	0.104	0.460
TMT-A _All_	18.47	-2.73 – 39.66	0.087	17.71	-8.06 – 43.48	0.176	0.540
TMT-A _NMDAR_	-12.97	-40.45 – 14.51	0.352	-12.56	-43.31 – 18.19	0.420	0.773
TMT-B _All_	0.97	-21.61 – 23.55	0.932	1.59	-24.44 – 27.63	0.904	0.963
TMT-B _NMDAR_	-6.10	-35.62 – 23.41	0.683	-8.09	-43.32 – 27.15	0.649	0.853
PSQI _All_	1.21	-0.11 – 2.54	0.073	1.08	-0.35 – 2.51	0.137	0.485
PSQI _NMDAR_	1.79	0.10 – 3.47	**0.038**	1.63	-0.08 – 3.34	0.062	0.407
EQ-5D-5L mobility _All_	0.64	0.15 – 1.14	**0.012**	0.51	0.04 – 0.99	**0.033**	0.407
EQ-5D-5L mobility _NMDAR_	1.17	0.55 – 1.79	**<0.001**	1.04	0.45 – 1.63	**0.001**	**0.046**
EQ-5D-5L self-care _All_	0.15	-0.29 – 0.58	0.505	0.08	-0.36 – 0.52	0.715	0.853
EQ-5D-5L self-care _NMDAR_	0.28	-0.28 – 0.84	0.319	0.19	-0.40 – 0.78	0.518	0.822
EQ-5D-5L activities _All_	0.40	-0.08 – 0.87	0.104	0.32	-0.14 – 0.78	0.173	0.540
EQ-5D-5L activities _NMDAR_	0.69	0.08 – 1.30	**0.027**	0.56	-0.02 – 1.14	0.059	0.407
EQ-5D-5L pain _All_	0.35	-0.13 – 0.83	0.149	0.25	-0.20 – 0.69	0.275	0.646
EQ-5D-5L pain _NMDAR_	0.65	0.03 – 1.28	**0.041**	0.44	-0.12 – 1.01	0.122	0.468
EQ-5D-5L anxiety _All_	0.27	-0.07 – 0.62	0.114	0.21	-0.14 – 0.57	0.239	0.644
EQ-5D-5L anxiety _NMDAR_	0.26	-0.18 – 0.71	0.244	0.18	-0.29 – 0.65	0.453	0.801
EQ-5D-5L today_All_	-8.55	-16.69 - -0.40	**0.040**	-6.04	-13.46 – 1.39	0.110	0.460
EQ-5D-5L today _NMDAR_	-13.21	-23.74 - -2.67	**0.014**	-9.06	-17.96 - -0.16	**0.046**	0.407
Max. walking speed _ALL_	-0.24	-0.50 – 0.02	0.065	-0.18	-0.40 – 0.04	0.107	0.460
Max. walking speed _NMDAR_	-0.38	-0.69 - -0.06	**0.020**	-0.30	-0.57 - -0.03	**0.027**	0.407
Max. O2 uptake _ALL_	0.06	-1.89 – 2.02	0.949	0.54	-1.41 – 2.50	0.582	0.853
Max. O2 uptake _NMDAR_	-0.68	-3.17 – 1.82	0.592	0.08	-2.41 – 2.57	0.949	0.970
FAC _ALL_	-0.42	-1.11 – 0.27	0.233	-0.40	-1.08 – 0.29	0.252	0.644
FAC _NMDAR_	0.24	-0.69 – 1.17	0.609	0.20	-0.65 – 1.05	0.636	0.853
6 min walk distance _ALL_	-27.12	-81.64 – 27.40	0.327	-15.48	-67.98 – 37.02	0.560	0.853
6 min walk distance _NMDAR_	-41.82	-111.57 – 27.93	0.237	-23.93	-91.25 – 43.38	0.482	0.821
Rivermead _ALL_	-0.28	-1.57 – 1.01	0.668	-0.23	-1.55 – 1.09	0.736	0.853
Rivermead _NMDAR_	0.01	-1.66 – 1.67	0.992	0.11	-1.49 – 1.72	0.888	0.963
Step count _ALL_	535.6	-1156.4 – 2227.7	0.531	533.79	-1037.1 – 2104.7	0.501	0.822
Step count _NMDAR_	-1348.1	-3701.4 – 1005.3	0.258	-958.35	-2861.8 – 945.1	0.319	0.646
REPAS _ALL_	-0.59	-4.33 – 3.14	0.753	-0.59	-4.09 – 2.91	0.739	0.853
REPAS _NMDAR_	-0.52	-5.41 – 4.38	0.835	-0.22	-4.66 – 4.22	0.921	0.963
Box and Block _ALL_	0.37	-5.90 – 6.65	0.906	0.06	-5.68 – 5.80	0.984	0.984
Box and Block _NMDAR_	2.49	-5.56 – 10.54	0.541	1.97	-5.51 – 9.44	0.603	0.853
MRC _ALL_	-0.30	-2.07 – 1.47	0.738	-0.27	-1.87 – 1.34	0.742	0.853
MRC _NMDAR_	0.54	-1.73 – 2.82	0.637	0.46	-1.55 – 2.47	0.653	0.853
Gait energy cost _ALL_	0.49	0.06 – 0.92	**0.028**	0.16	-0.18 – 0.51	0.349	0.669
Gait energy cost _NMDAR_	0.49	0.06 – 0.92	**0.028**	0.21	-0.21 – 0.62	0.323	0.646

*95% CI* 95% Confidence Interval; *mRS* modified Rankin scale; *BI* Barthel Index; *CES-D* Center for Epidemiologic Studies Depression scale; *MoCA* Montréal Cognitive Assessment; *TMT-A* Trail Making Test A; *TMT-B* Trail Making Test-B; *PSQI* Pittsburgh sleep quality index; *FAC* functional ambulation category; *REPAS* resistance to passive movement scale sum score; *MRC* medical research council scale for muscle strength, sum score over 6 items

Bold values indicate statistical significance (p < 0.05).

## 4 Discussion

The current study investigated the trajectory of neuronal autoantibodies detected in patient serum following ischemic or hemorrhagic stroke and their association with various outcome parameters in the subacute to chronic phase of stroke. To this purpose, we conducted comprehensive serial neuronal autoantibody profiling and assessed their association on multiple outcome domains at four timepoints over the course of six months post-stroke. We observed a divergent development of neuronal autoantibody prevalence, with cell-based autoantibody frequency decreasing and tissue-based frequency increasing with time. NMDAR was the most frequently detected autoantibody; however, seroreactivity in cell-based assays was identified for 11 additional neuronal autoantibodies. Due to the low number of patients positive for these antibodies, a comprehensive assessment of their clinical relevance was not possible. Patients with NMDAR antibodies at baseline were more likely to have a history of cerebrovascular disease, higher baseline triglyceride levels and lower baseline mRS scores compared to seronegative patients. Lastly, we found that patients with neuronal autoantibodies at baseline had worse outcomes in the CES-D depression score and poorer subjective mobility six months after stroke. NMDAR antibodies at baseline were associated with a lower subjective overall health rating and lower maximum walking speed at six months post-stroke. Interestingly, there was no statistically significant association with serostatus on outcome measures at three months following stroke.

The cause of the divergent trajectories of autoantibody prevalence in cell-based and tissue-based assays is not entirely clear. Both assays detect different, yet even partially overlapping antibody panels and the overall number of patients showing reactivity in tissue-based assays is lower than those showing reactivity in cell-based assays six months after stroke. The observed decline in reactivity over time in cell-based assays may reflect a transient, acute-phase immune response to exposed neuronal surface antigens following stroke, which potentially diminishes as stroke-induced immunosuppression causes a reduction of humoral autoimmunity. Conversely, the increase in tissue-based reactivity may indicate a chronic autoimmune or inflammatory response as well as processes of recovery in the six months following stroke.

The seroprevalence of neuronal autoantibodies in our cohort aligns with findings from previous studies. Serum NMDAR antibodies are the most common neuronal autoantibody in stroke, detected in 10% - 24% of stroke patients (6–10, 14, 15, 17). The statistically significantly higher prevalence of NMDAR antibodies (i.e., 44%) reported by Kalev-Zylinska et al. is likely attributed to their use of ELISA and Western blot techniques for antibody detection (18). In line with literature, NMDAR-IgM and -IgA isotypes were statistically significantly more prevalent than IgG in our cohort (7–9, 14–17). While NMDAR-IgG is generally regarded as pathogenic, the clinical significance of NMDAR-IgM and -IgA isotypes remains uncertain (6). These isotypes have been detected in both healthy and diseased individuals and are generally considered markers of physiological immune activity (6). NMDAR antibodies are linked to cognitive impairment across several neurological and systemic disorders (11–13). In stroke, their potential protective or detrimental effects are still unclear and may depend on blood-brain-barrier integrity (6, 7). There are limited data on the long-term dynamic of antibody prevalence following stroke (8, 18). The study by Kalev-Zylinska et al. reports an overall decline in NMDAR seropositivity at 18 ± 6 months post-stroke, which is comparable to our finding in cell-based assays (18). Similar to the findings of Royl et al., we also observed only a low rate of seroconversion after stroke (8). The prevalence of non-NMDAR antibodies in stroke patients is seldom reported in the literature. In the study by Royl et al., CASPR2 IgG was identified as the second most prevalent antibody; in our cohort this antibody was the fifth most frequent antibody (8). However, there was no additional overlap between the non-NMDAR antibodies we detected and those reported in their study (8). The study by Sperber et al. included one patient with LGI1 antibodies (titer 1:10) in a cohort of 583 patients (14, 17). These antibodies were not detected in our study. To our knowledge, tissue-reactivity patterns have not been reported in previous studies of stroke patients.

We found that patients with NMDAR antibodies were statistically significantly more likely to have reported a history of cerebrovascular disease, consistent with previous reports (8, 16); however, this association was largely attributable to a higher frequency of transient ischemic attacks. The overall percentage of history of stroke was numerically higher in patients with NMDAR-autoantibodies at baseline; nonetheless, this difference did not reach statistical significance. Transient ischemic attacks lack definitive diagnostic criteria and can occasionally be challenging to differentiate from other transient neurological defects; therefore our finding must be interpreted with caution. This possible association of a more frequent history of cerebrovascular disease is particularly interesting in adjunction to our finding, that cell-based seroprevalence decreases within six months after stroke. More studies are required to corroborate this finding; however, one possible explanation for this paradoxical observation is that patients with a history of cerebrovascular disease are more likely to be in a latent state of (low-grade) neuroinflammation, which promotes subsequent antibody production. In contrast, acute stroke induces immune system shifts including immunosuppression that may lead to antibody clearance with time. Additionally, we observed that patients with NMDAR antibodies have higher baseline triglyceride levels, a trend also reported in previous studies (9, 16). A possible explanation for this association is that elevated triglyceride levels are linked to an increased risk of atherosclerosis which has a strong inflammatory component (25). In contrast to Sperber et al.’s study, we found no significant differences in the occurrence of serious adverse events between seropositive and seronegative patients over the course of the study (9).

We confirmed findings that patients with neuronal autoantibodies had worse depression scores (17). In the study by Sperber et al., outcome was assessed 1–3 years post-stroke via telephone calls (17). In our trial, the association was already evident in clinical study visits at six, but not three months after stroke. However, the observed effect did not reach the threshold of the minimally clinical important difference (26). We observed poorer subjective mobility scores in the EQ-5D-5L in patients with neuronal autoantibodies. When regarding only patients with NMDAR antibodies we additionally found a lower subjective overall health rating in the EQ-5D-5L as well as a lower maximum walking speed at six months post-stroke. To our knowledge, these findings have not been previously reported and warrant further confirmation.

## 5 Strengths and limitations

The *PHYS-STROKE* cohort represents a high-quality, randomized-controlled multicenter trial which included patients with a relatively high clinical stroke severity. A clear strength of the study is the range of motor and non-motor outcome measures and thorough antibody profiling in cell- and tissue-based assays at multiple timepoints in the subacute and chronic phase of stroke. The current study has several limitations. One main consideration when interpreting our results is the large time window for the baseline blood draw (5–45 days post-stroke). This results in a heterogeneous patient population containing both patients with pre-existing neuronal autoantibodies and those who putatively have undergone seroconversion as a consequence of stroke. This is an important limitation, as the immune response following stroke is highly complex and dynamic and previous studies have shown that neuronal autoantibody prevalence varies considerably during the acute and subacute phases of stroke (7, 18). Notably, the comparable median baseline sampling times between seropositive and seronegative patients did not drive the observed differences in neuronal autoantibody prevalence in our cohort. Our patient cohort is heterogenous and includes patients with ischemic and hemorrhagic stroke and strokes in various territories. This limitation was particularly evident in our regression models, which revealed that the statistically significant outcomes were driven by ischemic stroke patients. It is important to note that the ischemic subgroup was substantially larger than the hemorrhagic stroke subgroup, which should be considered when interpreting these results. We employed the NIHSS to assess neurological deficits, recognizing that this scale was originally validated for ischemic stroke patients and may insufficiently capture certain neurological deficits more specific to hemorrhagic stroke. Nonetheless, it remains the most widely used assessment tool in both research and clinical practice across all stroke subtypes. As no established or validated alternative scoring systems exist specifically for hemorrhagic stroke, we believe its use in our mixed stroke population is justified. We did not incorporate by-proxy markers of blood-brain barrier integrity in our analysis. Although there is considerable overlap, the underlying population at the four study timepoints are not identical. To account for this, we replicated our results in patients with antibody measurements available at all timepoints (**table S4**) and addressed the reduced sample size over time using multiple imputation.

## 6 Conclusion

In our study, we were able to corroborate previous findings that showed a link between neuronal autoantibody seropositivity and poorer outcome in depression scores in patients with stroke. Additionally, we discovered associations between neuronal autoantibody seropositivity at baseline and neuropsychological and mobility outcomes six months post-stroke, suggesting a potential role of autoimmunity in motor and non-motor recovery following stroke. Further research with larger, pooled datasets and longer follow-up periods is necessary to confirm these findings.

## Data Availability

The datasets presented in this study can be found in online repositories. The names of the repository/repositories and accession number(s) can be found below: https://doi.org/10.5281/zenodo.15791897.
