# Supplementary material for: Neuronal autoantibodies associated with poorer neuropsychological and motor outcomes 6 months after stroke: results from the PHYS-STROKE trial
Source: Front Immunol. 2026 Mar 18;16:1678840. doi: 10.3389/fimmu.2025.1678840 (PMC13038563; doi:10.3389/fimmu.2025.1678840)
Supplement: Supplementary file 1 [file DataSheet1.docx]

**Supplementary Material to:**

**Neuronal autoantibodies associated with poorer neuropsychological and motor outcomes 6 months after stroke: Results from the *PHYS-STROKE* trial**

Charlotte Pietrock, Konrad Neumann, Kristin Rentzsch, Harald Prüss, Andreas Meisel, Matthias Endres, Alexander Heinrich Nave

**Table S1** | Inclusion and exclusion criteria of the *PHYS-STROKE* trial

| **Inclusion Criteria** | | |
| --- | --- | --- |
| 1. | Diagnosis of stroke (within 5-45 days post-stroke); ischaemic or haemorrhagic (cortical, subcortical, brainstem), as determined by initial magnetic resonance imaging (MRI) or computer tomography (CT) scan of the brain |  |
| 2. | Age ≥ 18 years |  |
| 3. | Ability to sit for ≥ 30 seconds (unsupported or supported by holding onto supports such as the edge of the bed) |  |
| 4. | Barthel Index ≤ 65 at inclusion |  |
| 5. | Considered able to perform aerobic exercise, as determined by the responsible physician |  |
| **Exclusion Criteria** | | |
| 1. | Patient considered unable to comply with study requirements |  |
| 2. | Stroke due to intracranial haemorrhage primarily due to bleeding from ruptured aneurysm or arteriovenous malformation |  |
| 3. | Progressive stroke |  |
| 4. | Unable to perform the required exercises due to a) medical, b) musculoskeletal, or c) neurological problems (details below, 4a-c) |  |
| 4a. | Medical problems: unstable cardiovascular condition or other serious cardiac conditions (e.g., New York Heart Association criteria for Class IV heart disease, hospitalization for myocardial infarction or heart surgery within 120 days, severe cardiomyopathy or documented serious and unstable cardiac arrhythmias) |  |
| 4b. | Musculoskeletal problems: restricted passive range of motion in the major lower limb joints (that is, an extension deficit of >20° for the affected hip or knee joints, or a dorsiflexion deficit of >20° for the affected ankle) |  |
| 4c. | Neurological problems: severity of stroke-related deficits |  |
| 5. | Required help of at least one person to walk before stroke due to neurological (e.g., advanced Parkinson’s disease, amyotrophic lateral sclerosis, multiple sclerosis) or non-neurological (e.g., heart failure, orthopaedic problems) co-morbidities |  |
| 6. | Life expectancy ≤ 1 year as determined by responsible physician |  |
| 7. | Drug or alcohol addiction within the last 6 months |  |
| 8. | Significant current psychiatric illness defined as affective disorder unresponsive to medication or bipolar affective disorder, psychosis, schizophrenia or suicidality |  |
| 9. | Current participation in another intervention trial |  |

**Table S2** | Sample characteristics of *PHYS-STROKE* outcome measures

|  | **Mean ± SD** | **Range** |
| --- | --- | --- |
| BL_mRS (*n* = 199) | 3.8 ± 0.4 | 2 - 4 |
| V2_mRS (*n* = 166) | 3.1 ± 0.9 | 1 - 4 |
| V3_mRS (*n* = 144) | 2.8 ± 1.0 | 0 - 4 |
| BL_BI (*n* = 199) | 47.6 ± 16.7 | 10 - 80 |
| V2_BI (*n* = 167) | 77.8 ± 20.8 | 10 – 100 |
| V3_BI (*n* = 144) | 82.9 ± 18.9 | 10 - 100 |
| BL_CESD (*n* = 180) | 17.1 ± 10.5 | 0 - 55 |
| V2_CESD (*n* = 153) | 14.4 ± 10.0 | 0 - 47 |
| V3_CESD (*n* = 138) | 12.5 ± 9.7 | 0 - 44 |
| BL_MoCA (*n* = 197) | 21.7 ± 6.9 | 0 - 30 |
| V2_MoCA (*n* = 163) | 23.5 ± 6.0 | 2 - 30 |
| V3_MoCA (*n* = 142) | 24.5 ± 5.8 | 2 - 30 |
| BL_TMT-A (s, *n* = 197) | 118.9 ± 92.2 | 19 - 301 |
| V2_TMT-A (s, *n* = 165) | 90.2 ± 84.3 | 17 - 301 |
| V3_TMT-A (s, *n* = 144) | 83.3 ± 81.8 | 15 - 301 |
| BL_TMT-B (s, *n* = 196) | 223.5 ± 88.1 | 47 - 301 |
| V2_TMT-B (s, *n* = 165) | 183.5 ± 95.4 | 36 - 301 |
| V3_TMT-B (s, *n* = 144) | 161.0 ± 91.7 | 36 - 301 |
| BL_PSQI (*n* = 183) | 4.4 ± 3.0 | 0 - 16 |
| V2_PSQI (*n* = 157) | 5.7 ± 3.4 | 0 - 15 |
| V3_PSQI (*n* = 137) | 4.7 ± 3.4 | 0 - 17 |
| BL_EQ5D5L _mobility_ (*n* = 193) | 3.5 ± 1.3 | 1 - 5 |
| V2_EQ5D5L _mobility_ (*n* = 164) | 2.9 ± 1.2 | 1 - 5 |
| V3_EQ5D5L _mobility_ (*n* = 144) | 2.6 ± 1.2 | 1 - 5 |
| BL_EQ5D5L _self-care_ (*n* = 193) | 2.9 ± 1.3 | 1 - 5 |
| V2_EQ5D5L _self-care_ (*n* = 164) | 2.2 ± 1.2 | 1 - 5 |
| V3_EQ5D5L _self-care_ (*n* = 144) | 2.1 ± 1.1 | 1 - 5 |
| BL_EQ5D5L _activities_ (*n* = 193) | 3.6 ± 1.3 | 1 - 5 |
| V2_EQ5D5L _activities_ (*n* = 163) | 2.9 ± 1.2 | 1 - 5 |
| V3_EQ5D5L _activities_ (*n* = 143) | 2.5 ± 1.2 | 1 - 5 |
| BL_EQ5D5L _pain_ (*n* = 193) | 2.2 ± 1.2 | 1 - 5 |
| V2_EQ5D5L _pain_ (*n* = 163) | 2.3 ± 1.1 | 1 - 5 |
| V3_EQ5D5L _pain_ (*n* = 144) | 2.1 ± 1.1 | 1 - 5 |
| BL_EQ5D5L _anxiety_ (*n* = 193) | 1.8 ± 1.0 | 1 - 5 |
| V2_EQ5D5L _anxiety_ (*n* = 163) | 1.7 ± 0.9 | 1 - 5 |
| V3_EQ5D5L _anxiety_ (*n* = 144) | 1.6 ± 0.9 | 1 - 5 |
| BL_EQ5D5L _today_ (*n* = 191) | 49.1 ± 21.9 | 0 - 100 |
| V2_EQ5D5L _today_ (*n* = 163) | 56.4 ± 21.7 | 0 - 100 |
| V3_EQ5D5L _today_ (*n* = 144) | 62.3 ± 20.1 | 1 - 100 |
| BL_RWT (*n* = 195) | 35.6 ± 18.4 | 0 - 86 |
| V2_RWT (*n* = 158) | 41.2 ± 18.0 | 0 - 81 |
| BL max. walking speed (m/s, *n* = 195) | 0.4 ± 0.4 | 0.0 – 2.5 |
| V2 max. walking speed (m/s, *n* = 163) | 0.8 ± 0.7 | 0.1 – 3.3 |
| V3 max. walking speed (m/s, *n* = 142) | 0.9 ± 0.8 | 0.1 – 5.0 |
| BL_O2-uptake (ml/kg/min, *n* = 171) | 21.8 ± 5.4 | 9.7 – 38.6 |
| V2_O2-uptake (ml/kg/min, *n* = 154) | 23.1 ± 6.2 | 0.6 – 46.9 |
| V3_O2-uptake (ml/kg/min, *n* = 139) | 23.6 ± 6.2 | 8.9 – 41.6 |
| BL_FAC (*n* = 199) | 1.7 ± 1.1 | 0 - 4 |
| V2_FAC (*n* = 95) | 3.1 ± 1.3 | 0 - 5 |
| V3_FAC (*n* = 79) | 3.5 ± 1.3 | 0 - 5 |
| BL_6min walk distance (m, *n* = 171) | 140.5 ± 110.9 | 4 - 670 |
| V2_6min walk distance (m, *n* = 155) | 201.3 ± 141.7 | 0 - 965 |
| V3_6min walk distance (m, *n* = 140) | 235.8 ± 150.8 | 0 - 603 |
| BL_RMI (*n* = 199) | 5.1 ± 2.8 | 0 - 14 |
| V2_RMI (*n* = 165) | 9.1 ± 4.1 | 0 - 15 |
| V3_RMI (*n* = 144) | 10.5 ± 3.9 | 0 - 15 |
| BL_actigraphy (steps/day, *n* = 184) | 4135.8 ± 3114.6 | 0 - 14112 |
| V2_actigraphy (steps/day, *n* = 146) | 5398.6 ± 3951.8 | 0 - 20641 |
| V3_actigraphy (steps/day, *n* = 115) | 5312.9 ±3283.9 | 0 - 14360 |
| BL_REPAS (*n* = 197) | 4.1 ± 5.0 | 0 - 20 |
| V2_REPAS (*n* = 162) | 7.5 ± 8.0 | 0 - 37 |
| V3_REPAS (*n* = 142) | 10.2 ± 10.6 | 0 - 39 |
| BL_BBT_impaired_ (blocks, *n* = 199) | 12.8 ± 16.3 | 0 - 63 |
| V2_BBT_impaired_ (blocks, *n* = 165) | 20.8 ± 20.0 | 0 - 70 |
| V3_BBT_impaired_ (blocks, *n* = 165) | 25.0 ± 21.5 | 0 - 69 |
| BL_MRC (*n* = 198) | 19.2 ± 8.3 | 0 - 30 |
| V2_MRC (*n* = 165) | 22.1 ± 6.5 | 0 - 30 |
| V3_MRC (*n* = 144) | 23.3 ± 6.0 | 3 - 30 |
| BL_EoG (ml/kg^-1^/m^-1^, *n* = 104) | 0.8 ± 0.7 | 0.1 – 3.5 |
| V2_EoG (ml/kg^-1^/m^-^1, *n* = 89) | 0.5 ± 0.4 | 0.1 – 2.3 |
| V3_EoG (ml/kg^-1^/m^-1^, *n* = 66) | 0.5 ± 0.5 | 0.1 – 2.6 |

*mRS* modified Rankin Scale; *BI* Barthel Index; *CESD* Center for Epidemiologic Studies Depression scale; *MoCA* Montréal Cognitive Assessment; *TMT-A* Trail Making Test A; *TMT-B* Trail Making Test-B; *PSQI* Pittsburgh sleep quality index; *RWT* Regensburg word fluency test; *FAC* functional ambulation category; *RMI* Rivermead mobility index; *REPAS* resistance to passive movement scale sum score; *BBT* Box and Block test, impaired hand; *MRC* medical research council scale for muscle strength, sum score over 6 items; *EoG* gait energy cost

**Table S3** | Neuronal autoantibodies included in panel

| **Abbreviation** | **Name** | |
| --- | --- | --- |
| ***Extracellular Antigens*** |  | |
| NMDAR | N-methyl-D-aspartate receptor |  |
| GABA-a | gamma-aminobutyric-acid A receptor |  |
| GABA-b | gamma-aminobutyric-acid B receptor |  |
| AQP4 | aquaporin 4 |  |
| LGI1 | leucine-rich glioma-inactivated 1 |  |
| CASPR2 | contactin-associated protein-like 2 |  |
| GluRD2 | glutamate receptor delta 2 |  |
| MOG | myelin oligodendrocyte glycoprotein |  |
| Flotillin | Flotillin1/2 |  |
| AMPAR1/2 | α-amino-3-hydroxy-5-methyl-4-isoxazolepropionic acid receptor 1/2 |  |
| GRM1 | Glutamate metabotropic receptor 1 |  |
| GRM5 | Glutamate metabotropic receptor 5 |  |
| GLRA1b | Glycine receptor α 1b |  |
| DRD2 | Dopamine receptor 2 |  |
| KCNA2 | Potassium voltage-gated channel subfamily A Member 2 |  |
| IgLON5 | IgLON family number 5 |  |
| DPPX | Dipeptidyl-peptidase-like protein 6 |  |
| AT1A3 | Sodium/potassium-transporting ATPase subunit α3 |  |
| Neurofascin 155 | Neurofascin 155 |  |
| Neurofascin 186 | Neurofascin 186 |  |
| CNTN1 | Contactin 1 |  |
| ERC1 | Acronym for previous protein names ELKS, RAB6IP2, CAST |  |
| Sez6l2 | Seizure-related 6 homolog like 2 antibodies |  |
| AP3B2 | Neuronale (B2) form of adaptor protein 3 (AP3) |  |
| CARPVIII | Carbonic anhydrase-related protein VIII |  |
| Neurochondrin | Neurochondrin |  |
| ITPR1 | Inositol 1,4,5-trisphosphate receptor 1 |  |
| Homer-3 | Homer protein homolog 3 |  |
| Neurexin 3α | Neurexin 3α |  |
| ARGHAP 26 | RhoGTPase-activating protein 26 |  |
| ***Intracellular Antigens*** |  |  |
| Amphiphysin | Amphiphysin |  |
| CV2 | Collapsing response mediator protein 5 |  |
| Yo | Yo |  |
| Ri | Ri |  |
| Ma2 | Ma-2 |  |
| Recoverin | Recoverin |  |
| Hu | Anna-1 |  |
| Zic4 | Zinc finger 4 |  |
| GAD65 | glutamic acid decarboxylase 65 |  |
| DNER | Delta/notch-like epidermal growth factor-related receptor |  |

**Table S4** | Antibody frequencies of patients with complete antibody analyses at all visits

|  | **Any timepoint (*n* = 109)** | **Baseline**  **(*n* = 109)** | **V1**  **(*n* = 109)** | **V2**  **(*n* = 109)** | **V3**  **(*n* = 109)** |
| --- | --- | --- | --- | --- | --- |
| **Time after stroke (days, median, Q1 25% - Q3 75%)** | - | 25 (16 – 37) | 56 (45 – 69) | 91 (87 – 94) | 180 (177 – 184) |
| **Tissue reactivity summarized (*n*, %)** | 9 (8.3) | 3 (2.8) | 5 (4.6) | 6 (5.5) | 7 (6.4) |
| **Cell-based seroreactivity summarized (all Igs, *n*, %)** | 32 (29.4) | 25 (22.9) | 21 (19.3) | 20 (18.3) | 18 (16.5) |
| **Cell-based seroreactivity summarized (only IgG, *n*, %)** | 16 (14.7) | 12 (11.0) | 8 (7.3) | 11 (7.3) | 8 (7.3) |
| **High-titer (≥1:100) cell-based seroreactivity summarized (all Igs, *n*, %)** | 17 (15.6) | 12 (11.0) | 10 (9.2) | 8 (7.3) | 7 (6.4) |
| **High-titer (≥1:100) cell-based seroreactivity summarized (only IgG, *n*, %)** | 8 (7.3) | 6 (5.5) | 6 (5.5) | 3 (2.8) | 2 (1.8) |
| **NMDAR Igs summarized (*n*, %)** | 18 (16.5) | 14 (12.8) | 13 (11.9) | 10 (9.2) | 12 (11.0) |
| NMDAR IgM (*n*, %) | 13 (11.9) | 9 (8.3) | 10 (9.2) | 8 (7.3) | 6 (5.5) |
| NMDAR IgA (*n*, %) | 10 (9.2) | 7 (6.4) | 7 (6.4) | 7 (6.4) | 8 (7.3) |
| NMDAR IgG (*n*, %) | 1 (0.9) | 1 (0.9) | 0 | 1 (0.9) | 1 (0.9) |
| **NMDAR high-titer (≥1:100), all Igs summarized (*n*, %)** | 9 (8.3) | 6 (5.5) | 4 (3.7) | 5 (4.6) | 5 (4.6) |
| Homer3 IgG (*n*, %) | 2 (1.8) | 2 (1.8) | 1 (0.9) | 0 | 0 |
| GLRA1b IgG (*n*, %) | 3 (2.8) | 2 (1.8) | 2 (1.8) | 3 (2.8) | 2 (1.8) |
| Flotillin IgG (*n*, %) | 2 (1.8) | 1 (0.9) | 1 (0.9) | 2 (1.8) | 1 (0.9) |
| CASPR2 IgG (*n*, %) | 2 (1.8) | 1 (0.9) | 0 | 1 (0.9) | 1 (0.9) |
| KCNA2 IgG (*n,* %) | 1 (0.9) | 1 (0.9) | 1 (0.9) | 1 (0.9) | 1 (0.9) |
| Neurochondrin IgG (*n*, %) | 1 (0.9) | 1 (0.9) | 1 (0.9) | 1 (0.9) | 0 |
| MOG IgG (*n*, %) | 1 (0.9) | 1 (0.9) | 1 (0.9) | 1 (0.9) | 1 (0.9) |
| AP3B2 IgG (*n*, %) | 1 (0.9) | 1 (0.9) | 0 | 1 (0.9) | 1 (0.9) |
| ITPR1 IgG (*n*, %) | 1 (0.9) | 0 | 1 (0.9) | 0 | 0 |
| GRM5 IgG (*n*, %) | 1 (0.9) | 0 | 0 | 0 | 1 (0.9) |
| DRD2 IgG (*n*, %) | 1 (0.9) | 1 (0.9) | 0 | 0 | 0 |

No reactivity found for: GAD65 IgG, GABA-b IgG, Aquaporin 4 IgG, LGI1 IgG, GluRD2 IgG, GABA-a IgG, AMPAR IgG, GRM1 IgG, Ma2 IgG, Recoverin IgG, Zic-4 IgG, Iglon 5 IgG, DPPX IgG, AT1A3 IgG, Neurofascin 155 IgG, Neurofascin 186 IgG, CNTN1 IgG, CV2 IgG, Hu IgG, Ri IgG, ERC1 IgG, Rho-GTPase 26 IgG, DNER IgG, Sez6I2 IgG, CARPVIII IgG, Amphiphysin IgG, Yo IgG, Neurexin 3-alpha IgG


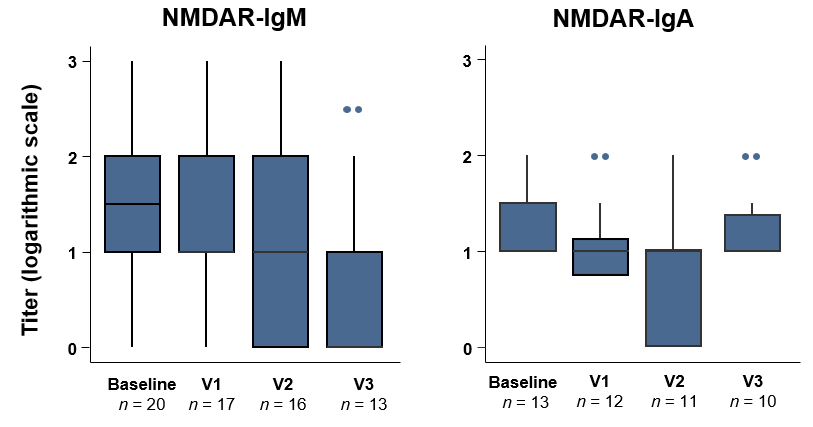


**Figure S1.** Development of titer over time on a logarithmic scale by NMDAR-isotype.

**Table S5** | Characteristics of seropositive patients

| **Antibody** | **MOG** | **KCNA 2** | **AP3B2** | **Neurochondrin** |
| --- | --- | --- | --- | --- |
| **Dynamic over Time** | 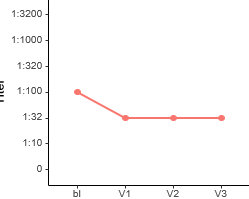 | 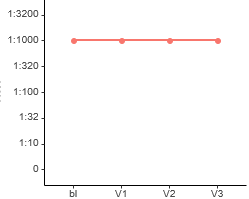 | 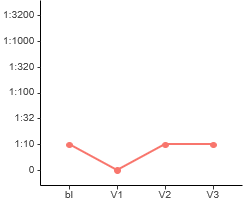 | 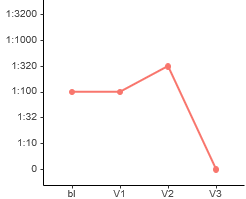 |
| **Titer over Time** | Baseline: 1:100  V1: 1:32  V2: 1:32  V3: 1:32 | Baseline: 1:1000  V1: 1:1000  V2: 1:1000  V3: 1:1000 | Baseline: 1:10  V1: 0  V2: 1:10  V3: 1:10 | Baseline: 1:100  V1: 1:100  V2: 1:320  V3: 0 |
| **Additional antibody reactivity** | - | Tissue-reactive | - | - |
| **Stroke type** | ischemic | ischemic | ischemic | ischemic |
| **TOAST** | cardioembolic | large-artery atherosclerosis | small-vessel occlusion | small-vessel occlusion |
| **Stroke localisation** | Left anterior circulation | Left anterior circulation | Right anterior circulation | Left anterior circulation |
| **Normalised FLAIR-based stroke lesion volume (ml)** | NA | NA | 9,0 | 9,8 |
| **NIHSS_baseline_** | 5 | 8 | 15 | 6 |
| **Barthel-Index _baseline_** | 55 | 60 | 35 | 90 |
| **mRS_baseline_** | 3 | 4 | 4 | 3 |
| **Previous cerebrovascular disease** | - | - | - | - |
| **Comorbidities** | Arterial hypertension | Arterial hypertension, coronary heart disease, peripheral artery disease | Arterial hypertension, Hypercholesterolemia, Diabetes mellitus, sleep apnoea | Arterial hypertension |
| **Medication prior to stroke** | Apixaban  Metoprolol  Kaliumcholoride  Fragmin P | ASS  Amlodipin  Candesartan  Escitalopram  Atorvastatin | ASS  Simvastatin  Metoprolol  Lisinopril  HCT  Sitagliptin  Metformin  Repaglinid  Citalopram  Clexane | ASS  Escitalopram  Metoprolol  Mirtazapin  Simvastatin  Delix plus (Ramipril/HCT)  Ramipril |

| **Antibody** | **GRM5** | **DRD2** | **CV2** | **ITPR1** |
| --- | --- | --- | --- | --- |
| **Dynamic over Time** | 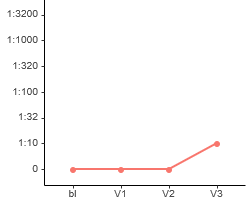 | 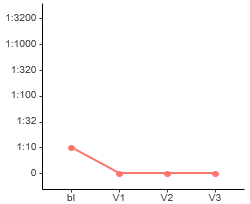 | 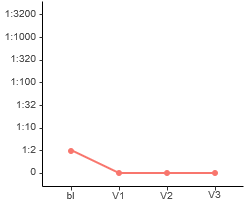 | 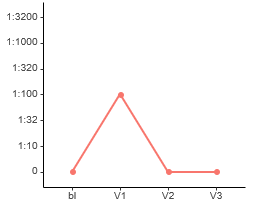 |
| **Titer over Time** | Baseline: 0  V1: 0  V2: 0  V3: 1:10 | Baseline: 1:10  V1: 0  V2: 0  V3: 0 | Baseline: 1:2  V1: 0  V2: 0  V3: 0 | Baseline: 0  V1: 1:100  V2: 0  V3: 0 |
| **Additional antibody reactivity** | GLRA1b  Baseline: 1:32  V1: 1:100  V2: 1:32  V3: 1:10 | - | - | Tissue-reactive (unspecific) |
| **Stroke type** | ischemic | ischemic | ischemic | ischemic |
| **TOAST** | cardioembolic | large-artery atherosclerosis | cardioembolic | Undetermined etiology |
| **Stroke localisation** | Left posterior circulation | Right anterior circulation | Right anterior circulation | Left anterior circulation |
| **Normalised FLAIR-based stroke lesion volume (ml)** | NA | 105,7 | NA | NA |
| **NIHSS_baseline_** | 6 | 11 | 4 | 4 |
| **Barthel-Index_baseline_** | 45 | 60 | 65 | 65 |
| **mRS_baseline_** | 4 | 3 | 3 | 3 |
| **Previous cerebrovascular disease** | - | stroke | - | - |
| **Comorbidities** | Arterial hypertension | Diabetes mellitus | Arterial hypertension, Atrial fibrillation, Diabetes mellitus | Arterial hypertension |
| **Medication prior to stroke** | Candesartan  Simvastatin  Apixaban  HCT  Folic acid  Pantoprazol  Movicol | ASS  Atorvastatin  Metformin | Enoxaparin  Pantoprazol  Amlodipin  Carvedilol  Apixaban  Dekristol  Vigantoletten | Atorvastatin  ASS  Ramipril  Enoxaparin |

| **Antibody** | **Homer 3** | | | |
| --- | --- | --- | --- | --- |
| **Dynamic over Time** | 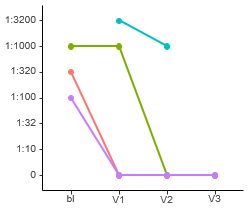 | | | |
| **Titer** | BL: 1:320  V1: 0  V2: NA  V3: NA | BL: 1:1000  V1: 1:1000  V2: 0  V3: 0 | BL: NA  V1: 1:3200  V2: 1:1000  V3: NA | BL: 1:100  V1: 0  V2: 0  V3: 0 |
| **Additional antibody reactivity** | none | none | BL: NA  V1: NMDA IgM 1:32  V2: NMDA-IgM 1:32  V3: NA | none |
| **Stroke type** | ischemic | ischemic | ischemic | haemorrhagic |
| **TOAST** | small-vessel occlusion | Undetermined etiology | large-artery atherosclerosis | / |
| **Stroke localisation** | Right posterior circulation | Right anterior circulation | Right anterior circulation | Left anterior circulation |
| **Normalised FLAIR-based stroke lesion volume (ml)** | 0,9 | 14,5 | NA | NA |
| **NIHSS_baseline_** | 9 | 4 | 13 | 5 |
| **Barthel-Index_baseline_** | 60 | 65 | 20 | 35 |
| **mRS_baseline_** | 3 | 3 | 4 | 4 |
| **Previous cerebrovascular disease** | none | none | none | none |
| **Comorbidities** | Arterial hypertension, Hypercholesterinaemia, coronary heart disease | Arterial hypertension | Arterial hypertension, Hypercholesterinaemia | Arterial hypertension, Diabetes mellitus |
| **Medication prior to stroke** | ASS, Enalapril, Simvastatin, Gabapentin, Acopt, Dekristol, Pantoprazol | ASS, Atorvastatin, Ramipril, Fluoxetin | ASS, Amlodipin, Atorvastatin, Torasemid, Fentanyl, Mirtazapin, Ramipril, Metoprolol, Tamsulosin, Novalgin | Ramipril, HCT, Nebivolol, Allopurinol |

| **Antibody** | **GLRA1b** | | | |
| --- | --- | --- | --- | --- |
| **Dynamic over Time** | 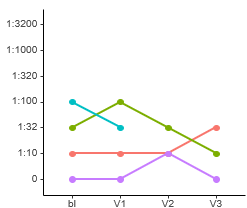 | | | |
| **Titer** | BL: 1:10  V1: 1:10  V2: 1.10  V3: 1:32 | BL: 1:32  V1: 1:100  V2: 1: 32  V3: 1:10 | BL: 1:100  V1: 1:32  V2: NA  V3: NA | BL: 0  V1: 0  V2: 1:10  V3:0 |
| **Additional antibody reactivity** | none | V3: GRM5 | none | none |
| **Stroke type** | haemorrhagic | ischemic | ischemic | ischemic |
| **TOAST** | NA | cardioembolic | Undetermined etiology | cardioembolic |
| **Stroke localisation** | Right anterior circulation | Left posterior circulation | Left anterior circulation | Right anterior circulation |
| **Normalised FLAIR-based stroke lesion volume (ml)** | NA | NA | 10,5 | 70,1 |
| **NIHSS_baseline_** | 8 | 6 | 12 | 12 |
| **Barthel-Index_baseline_** | 40 | 45 | 30 | 15 |
| **mRS_baseline_** | 4 | 4 | 4 | 4 |
| **Previous cerebrovascular disease** | stroke | none | none | stroke |
| **Comorbidities** | Arterial hypertension, Hypercholesterinaemia, coronary heart disease, atrial fibrillation | Arterial hypertension | Arterial hypertension, Hypercholesterinaemia, Diabetes mellitus, | Arterial hypertension, atrial fibrillation, Diabetes mellitus |
| **Medication prior to stroke** | Amlodipin, Valsartan, Bisoprolol, Doxazosin, Indapamid, Pantoprazol, Simvastatin, Fraxiparin | Candesartan, Simvastatin, HCT, Folic acid, Pantoprazol, Movicol, Apixaban | ASS, Atorvastatin, Ramipril, Pantoprazol | Metformin, Dabigatran |

| **Antibody** | **Flotillin** | | |
| --- | --- | --- | --- |
| **Dynamic over Time** | 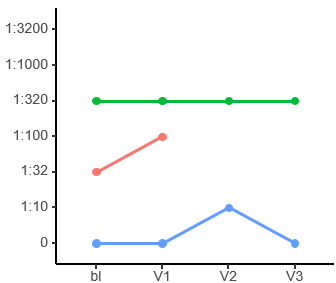 | | |
| **Titer** | BL: 1:32  V1: 1:100  V2: NA  V3: NA | BL: 1:320  V1: 1:320  V2: 1:320  V3: 1:320 | BL: 0  V1: 0  V2: 1:10  V3: 0 |
| **Additional antibody reactivity** | none | BL, V1, V2, V3: Tissue reactive: Flotillin-pattern (1:100) | none |
| **Stroke type** | ischemic | ischemic | Haemorrhagic |
| **TOAST** | large-artery atherosclerosis | large-artery atherosclerosis | / |
| **Stroke localisation** | Left anterior circulation | Right anterior circulation | Right anterior circulation |
| **Normalised FLAIR-based stroke lesion volume (ml)** | NA | NA | NA |
| **NIHSS_baseline_** | 7 | 7 | 7 |
| **Barthel-Index_baseline_** | 65 | 45 | 50 |
| **mRS_baseline_** | 2 | 4 | 3 |
| **Previous cerebrovascular disease** | none | none | none |
| **Comorbidities** | Arterial hypertension, coronary heart disease, peripheral artery disease | Arterial hypertension, Hypercholesterinaemia, Diabetes mellitus | None documented |
| **Medication prior to stroke** | ASS, Torasemid, Metoprolol, Olmesartan, HCT, Amlodipin, Duloxetin, Atorvastatin, Febuxostat, Fraxiparin | ASS, Metformin, Simvastatin, Tamsulosin | Levetiractam, Marcumar |

| **Antibody** | **CASPR2** | | |
| --- | --- | --- | --- |
| **Dynamic over Time** | 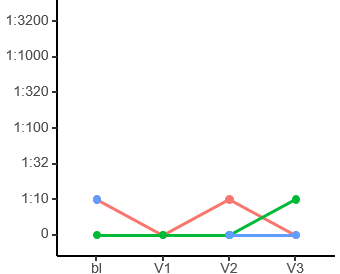 | | |
| **Titer** | BL: 1:10  V1: 0  V2: 1:10  V3: 1:10 | BL: 0  V1: 0  V2: 0  V3: 1:10 | BL: 1:10  V1: NA  V2: 0  V3: 0 |
| **Additional antibody reactivity** | none | BL, V1, V2, V3: NMDA IgA: 1:100 | none |
| **Stroke type** | ischemic | ischemic | ischemic |
| **TOAST** | large-artery atherosclerosis | large-artery atherosclerosis | small-vessel occlusion |
| **Stroke localisation** | Left posterior circulation | Right anterior circulation | Right anterior circulation |
| **Normalised FLAIR-based stroke lesion volume (ml)** | NA | 35,3 | 9,5 |
| **NIHSS_baseline_** | 7 | 6 | 4 |
| **Barthel-Index_baseline_** | 60 | 30 | 35 |
| **mRS_baseline_** | 3 | 4 | 4 |
| **Previous cerebrovascular disease** | none | TIA | none |
| **Comorbidities** | Arterial hypertension, coronary heart disease | Arterial hypertension, Hypercholesterinaemia, coronary heart disease | Arterial hypertension, Diabetes mellitus |
| **Medication prior to stroke** | ASS, Allopurinol, Atorvastatin, Clopidogrel, Ramipril, Heparin | ASS, Clopidogrel, Atorvastatin, Bisoprolol, Nitrospray, Ramipril, Indapamid, Pantoprazol, Mirtazapin, Fraxiparin | ASS, Candesartan, Metformin, Brinzolamid, Travaniprost, Simvastatin, Mirtazapin |

| **Antibody** | **NMDAR IgG** | |
| --- | --- | --- |
| **Dynamic over Time** | 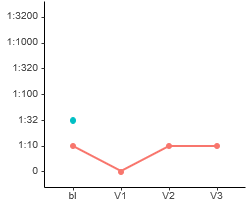 | |
| **Titer** | BL: 1:10  V1: 0  V2: 1:10  V3: 1:10 | BL: 1:32  V1: NA  V2: NA  V3: NA |
| **Additional antibody reactivity** | BL: NMDA IgA: 1:100; NMDA IgM: 1:32  V1: NMDAIgA: 1:10; NMDA IgM: 1:10  V2: NMDA IgA: 1:10; NMDA IgM: 1:10  V3: NMDA IgM: 1:10 | none |
| **Stroke type** | ischemic | ischemic |
| **TOAST** | small-vessel occlusion | cardioembolic |
| **Stroke localisation** | Right-hemispheric | Right anterior circulation |
| **Normalised FLAIR-based stroke lesion volume (ml)** | 5,6 | NA |
| **NIHSS_baseline_** | 6 | 18 |
| **Barthel-Index_baseline_** | 65 | 35 |
| **mRS_baseline_** | 3 | 4 |
| **Previous cerebrovascular disease** | none | TIA |
| **Comorbidities** | Arterial hypertension,Hypercholesterinaemia, Diabetes mellitus | Arterial hypertension, atrial fibrillation |
| **Medication prior to stroke** | Metformin, Sitagliptin, ASS, Simvastatin, Ramipril, Metamizol, Ibuprofen, Pregabalin, Insulin Humalog, Lantus, | Apixaban, Nebivolol, Torasemid, Valsartan, Atorvastatin |
| **Additional Info** |  | Patient only completed BL, is afterwards listed as “drop out” reasons unclear |

Characteristics of patients seropositive for NMDAR IgG and 12 further (non-NMDAR) neuronal autoantibodies. Each column represents one individual patient.

**Table S6** | Baseline Characteristics of *PHYS-STROKE* patients

|  | **Total**  **(*n* = 168)** | **NMDAR-seroreactive at baseline**  **(*n* = 24)** | **Seronegative for all antibodies at baseline**  **(*n* = 144)** | ***p*-value** |
| --- | --- | --- | --- | --- |
| **Baseline characteristics** |  |  |  |  |
| Demographic information |  |  |  |  |
| Age (years, mean ± SD) | 69.0 ± 11.6 | 71.4 ± 12.7 | 68.6 ± 11.4 | 0.158 |
| Patients aged ≥ 76 years (*n*, %) | 54 (32.1) | 10 (41.7) | 44 (30.6) | 0.447 |
| Female (*n*, %) | 68 (40.5) | 14 (58.4) | 54 (37.5) | 0.089 |
| Time from stroke to baseline blood draw (days, median, Q1 25% - Q2 75%) | 25 (15.8 - 36.3) | 17 (13.8 - 30.5) | 26 (16.8 - 37.0) | 0.159 |
| Stroke characteristics |  |  |  |  |
| Ischemic stroke (*n*, %) | 152 (90.5) | 23 (95.8) | 129 (89.6) | 0.555 |
| TOAST (*n*, %); 17 missing |  |  |  |  |
| Large-artery atherosclerosis | 27 (17.9) | 7 (30.4) | 20 (15.6) | 0.158 |
| Cardioembolic | 31 (20.5) | 5 (21.7) | 26 (20.3) | 1.000 |
| Small-vessel occlusion | 23 (15.2) | 2 (8.7) | 21 (16.4) | 0.527 |
| Other determined etiology | 6 (4.0) | 1 (4.3) | 5 (3.9) | 1.000 |
| Undetermined etiology | 55 (36.4) | 6 (26) | 49 (38.3) | 0.377 |
| Two or more etiologies | 9 (6.0) | 2 (8.7) | 7 (5.5) | 0.902 |
| NIHSS (median, Q1 25% - Q2 75%);  1 missing | 8 (5 - 12) | 7 (6 - 12.3) | 8 (5 - 12) | 0.871 |
| mRS (median, Q1 25% - Q2 75%) | 4 (4 - 4) | 4 (3 - 4) | 4 (4 - 4) | **0.011** |
| BI (median, Q1 25% - Q2 75%) | 50 (35 - 60) | 52.5 (35 - 65) | 50 (35 - 60) | 0.810 |
| Normalised lesion volume at baseline (ml, mean ± SD) | *n* = 79  47.4 ± 70.2 | *n* = 13  58.9 ± 73.4 | *n* = 66  45.1 ± 70.0 | 0.434 |
| Normalised lesion volume at V1 (ml, mean ± SD) | *n* = 62  40.7 ± 63.4 | *n* = 11  56.2 ± 85.4 | *n* = 51  37.3 ± 58.1 | 0.765 |
| Difference in mean lesion volume (V1-baseline, ml) | *n* = 57  -3.2 | *n* = 11  -2.4 | *n* = 46  -3.4 | 0.960 |
| Affected hemisphere (*n*, %); 2 missing |  |  |  |  |
| Left | 70 (42.2) | 9 (37.5) | 61 (43.0) | 0.782 |
| Right | 96 (57.8) | 15 (62.5) | 81 (47.0) | 0.782 |
| Affected circulation (*n*, %) |  |  |  |  |
| Anterior circulation | 129 (76.8) | 19 (79.2) | 110 (76.4) | 0.970 |
| Posterior circulation | 40 (23.8) | 5 (20.8) | 35 (24.3) | 0.912 |
| Cardiovascular risk factors |  |  |  |  |
| History of cerebrovascular disease (stroke and/or TIA; *n*, %) | 50 (29.8) | 12 (50) | 38 (26.4) | **0.036** |
| History of stroke (*n*, %) | 34 (20.2) | 7 (29.2) | 27 (18.8) | 0.367 |
| History of TIA (*n*, %) | 22 (13.1) | 7 (29.2) | 15 (10.4) | **0.028** |
| History of atrial fibrillation (*n*, %) | 42 (25.0) | 7 (29.2) | 35 (24.3) | 0.799 |
| History of arterial hypertension (*n*, %) | 142 (84.5) | 21 (87.5) | 121 (84.0) | 0.896 |
| History of hypercholesterinaemia (*n*, %) | 67 (39.9) | 13 (54.2) | 54 (37.5) | 0.187 |
| History of diabetes mellitus (*n*, %) | 55 (32.7) | 8 (33.4) | 47 (32.6) | 1.000 |
| History of myocardial infarction (*n*, %) | 2 (1.2) | 0 | 2 (1.4) | 1.000 |
| History of sleep apnoea (*n*, %) | 3 (1.8) | 0 | 3 (2.1) | 1.000 |
| History of malignant disease (*n*, %) | 17 (10.1) | 2 (8.4) | 15 (10.4) | 1.000 |
| History of smoking (*n*, %); 4 missing | 81 (48.2) | 11 (45.8) | 70 (48.6) | 1.000 |
| History of smoking (*n*, %) | 52 (31.0) | 9 (37.5) | 43 (29.9) | 0.609 |
| Pack-years (years, mean ± SD) | 38.7 ± 31.2 | 34.6 ± 27.6 | 39.7 ± 32.3 | 0.829 |
| BMI (kg/m^2^, mean ± SD) | 26.0 ± 4.1 | 26.3 ± 5.2 | 26.0 ± 3.9 | 0.844 |
| HbA1c (%, mean ± SD) | 6.1 ± 1.2 | 6.2 ± 1.0 | 6.1 ± 1.2 | 0.417 |
| Triglyceride (mmol/l, mean ± SD) | 1.4 ± 0.6 | 1.7 ± 0.5 | 1.4 ± 0.6 | **0.002** |
| HDL-Cholesterol (mmol/l, mean ± SD) | 1.3 ± 0.3 | 1.1 ± 0.4 | 1.3 ± 0.3 | 0.064 |
| LDL-Cholesterol (mmol/l, mean ± SD) | 2.3 ± 0.9 | 2.3 ± 0.8 | 2.3 ± 0.9 | 0.815 |
| Lipoprotein(a) (mg/dl, mean ± SD); 11 missing | 47.2 ± 55.2 | 35.1 ± 38.7 | 49.4 ± 57.5 | 0.464 |
| hs-CRP (mg/l, mean ± SD); 5 missing | 12.0 ± 18.4 | 13.9 ± 21.0 | 11.7 ± 17.9 | 0.294 |
| White Blood Cells (/nl, mean ± SD) | 7.3 ± 1.9 | 7.4 ± 1.7 | 7.3 ± 2.0 | 0.861 |

*TOAST* Trial of Org. 10172 in Acute Stroke Treatment; *NIHSS* National Institutes of Health Stroke Scale; *mRS* modified Rankin Scale; *BI* Barthel Index; *TIA* Transient ischemic attack; *BMI* Body Mass Index; *hs-CRP* high-sensitive C-reactive protein

**Table S7** | Serious adverse events

|  | **Total (*n* = 168)** | **NMDAR-seroreactive at baseline (*n* = 24)** | **Seronegative for all antibodies at baseline (*n* = 144)** | ***p*-value** |
| --- | --- | --- | --- | --- |
| Patients with any number of SAE within 6 months (*n*, %) | 34 (20.2) | 4 (16.7) | 30 (20.8) | 0.845 |
| Patients with >1 SAE within 6 months (*n*, %) | 8 (4.8) | 2 (8.3) | 6 (4.2) | 0.712 |
| Death within 6 months (*n*, %) | 5 (3.0) | 0 (0) | 5 (3.5) | 0.781 |
| Cardiovascular event within 6 months (*n*, %) | 0 (0) | 0 (0) | 0 (0) | - |
| Cerebrovascular event within 6 months (*n*, %) | 11 (6.5) | 2 (8.3) | 9 (6.3) | 1.000 |
| Referral to hospital (*n*, %) | 23 (13.7) | 3 (12.5) | 20 (13.9) | 1.000 |

*SAE* serious adverse event

**Table S8** | Factors associated with autoantibody seropositivity in tissue-based analyses over time

|  | **Original Model** | | | | **Imputed Model** | | | | | | | | | | |
| --- | --- | --- | --- | --- | --- | --- | --- | --- | --- | --- | --- | --- | --- | --- | --- |
|  | **OR** | **95% CI** | ***p*-value** | | **OR** | **95% CI** | | | | ***p*-value** | | | ***Adjusted***  ***p-value*** | | |
| Time (reference: baseline) |  | | | |  | |  |  | | |  | | | |  |
| Post-intervention (V1) | 1.99 | 0.85 – 4.65 | | 0.112 | 2.05 | 0.78 – 5.43 | | | | 0.148 | | | 0.444 | | |
| 3 months post-stroke (V2) | 2.19 | 0.95 – 5.08 | | 0.066 | 2.24 | 0.83 – 6.10 | | | | 0.114 | | | 0.444 | | |
| 6 months post-stroke (V3) | 3.21 | 1.33 – 7.72 | | **0.009** | 3.07 | 1.11 – 8.51 | | | | **0.032** | | | 0.288 | | |
| Age group (reference: ≤ 65 yrs) |  | | | |  | |  | |  | | |  | |  |  |
| Age 66 – 75 yrs | 1.26 | 0.31 – 5.13 | | 0.746 | 1.16 | 0.33 – 4.08 | | | | 0.815 | | | 0.917 | | |
| Age ≥ 76 yrs | 1.06 | 0.20 – 5.60 | | 0.944 | 0.96 | 0.22 – 4.25 | | | | 0.958 | | | 0.958 | | |
| Sex (reference: male) | 1.24 | 0.34 – 4.49 | | 0.746 | 1.21 | 0.38 – 3.88 | | | | 0.746 | | | 0.917 | | |
| Intervention (reference: fitness) | 1.22 | 0.37 – 3.97 | | 0.745 | 1.13 | 0.39 – 3.26 | | | | 0.815 | | | 0.917 | | |
| NIHSS_baseline_ | 0.98 | 0.91 – 1.06 | | 0.594 | 0.96 | 0.88 – 1.05 | | | | 0.409 | | | 0.842 | | |
| History of cerebrovascular disease (reference: negative) | 0.59 | 0.11 – 3.13 | | 0.532 | 0.60 | 0.15 – 2.40 | | | | 0.468 | | | 0.842 | | |

*OR* Odds Ratio; *95% CI* 95% Confidence Interval; *NIHSS* National Institutes of Health Stroke Scale.

**Table S9** | Influence of neuronal autoantibodies (all or NMDAR only) on outcome measures at 6 months post-stroke (crude models)

|  | **Original Model** | | |
| --- | --- | --- | --- |
|  | **ß** | **95% CI** | ***p*-value** |
| mRS _All_ | -0.07 | -0.53 – 0.38 | 0.747 |
| mRS _NMDAR_ | -0.09 | -0.68 – 0.51 | 0.775 |
| BI _All_ | -3.65 | -11.70 – 4.40 | 0.371 |
| BI _NMDAR_ | 0.09 | -10.35 – 10.53 | 0.987 |
| CES-D _All_ | 3.84 | -0.51 – 8.19 | 0.083 |
| CES-D _NMDAR_ | 3.91 | -1.55 – 9.38 | 0.159 |
| MoCA _All_ | -0.26 | -2.72 – 2.20 | 0.833 |
| MoCA _NMDAR_ | 1.47 | -1.65 – 4.59 | 0.353 |
| TMT-A _All_ | 25.55 | -8.83 – 59.93 | 0.144 |
| TMT-A _NMDAR_ | -23.54 | -68.16 – 21.09 | 0.299 |
| TMT-B _All_ | 11.67 | -27.93 – 51.26 | 0.561 |
| TMT-B _NMDAR_ | -21.07 | -72.19 – 30.06 | 0.416 |
| PSQI _All_ | 1.05 | -0.43 – 2.54 | 0.163 |
| PSQI _NMDAR_ | 1.48 | -0.40 – 3.36 | 0.123 |
| EQ-5D-5L mobility _All_ | 0.47 | -0.04 – 0.98 | 0.069 |
| EQ-5D-5L mobility _NMDAR_ | 0.95 | 0.30 – 1.59 | **0.004** |
| EQ-5D-5L self-care _All_ | 0.02 | -0.43 – 0.47 | 0.925 |
| EQ-5D-5L self-care _NMDAR_ | 0.09 | -0.49 – 0.68 | 0.753 |
| EQ-5D-5L activities _All_ | 0.13 | -0.39 – 0.64 | 0.622 |
| EQ-5D-5L activities _NMDAR_ | 0.43 | -0.23 – 1.09 | 0.201 |
| EQ-5D-5L pain _All_ | 0.47 | -0.01 – 0.96 | 0.056 |
| EQ-5D-5L pain _NMDAR_ | 0.74 | 0.12 – 1.37 | **0.019** |
| EQ-5D-5L anxiety _All_ | 0.28 | -0.09 – 0.65 | 0.137 |
| EQ-5D-5L anxiety _NMDAR_ | 0.23 | -0.25 – 0.71 | 0.352 |
| EQ-5D-5L today_All_ | -8.54 | -17.24 – 0.15 | 0.054 |
| EQ-5D-5L today _NMDAR_ | -11.40 | -22.63 – -0.18 | **0.047** |
| Max. walking speed _ALL_ | -0.10 | -0.47 – 0.28 | 0.606 |
| Max. walking speed _NMDAR_ | -0.17 | -0.63 – 0.30 | 0.479 |
| Max. O2 uptake _ALL_ | -1.49 | -4.20 – 1.23 | 0.280 |
| Max. O2 uptake _NMDAR_ | -1.87 | -5.32 – 1.58 | 0.286 |
| FAC _ALL_ | -0.48 | -1.23 – 0.27 | 0.207 |
| FAC _NMDAR_ | 0.20 | -0.80 – 1.21 | 0.686 |
| 6 min walk distance _ALL_ | -27.37 | -94.24 – 39.51 | 0.420 |
| 6 min walk distance _NMDAR_ | -31.33 | -116.39 – 53.74 | 0.468 |
| Rivermead _ALL_ | -0.59 | -2.29 – 1.12 | 0.497 |
| Rivermead _NMDAR_ | -0.04 | -2.25 – 2.16 | 0.969 |
| Step count _ALL_ | 389.81 | -1374.13 – 2153.76 | 0.662 |
| Step count _NMDAR_ | -1377.92 | -3755.69 – 999.84 | 0.253 |
| REPAS _ALL_ | -1.25 | -5.93 – 3.42 | 0.597 |
| REPAS _NMDAR_ | -0.11 | -6.26 – 6.05 | 0.972 |
| Box and Block _ALL_ | -0.94 | -10.25 – 8.37 | 0.842 |
| Box and Block _NMDAR_ | -0.34 | -12.37 – 11.70 | 0.956 |
| MRC _ALL_ | 0 | -2.63 – 2.62 | 0.997 |
| MRC _NMDAR_ | 1.08 | -2.30 – 4.47 | 0.528 |
| Gait energy cost _ALL_ | 0.14 | -0.24 – 0.52 | 0.457 |
| Gait energy cost _NMDAR_ | 0.20 | -0.25 – 0.65 | 0.381 |

*95% CI* 95% Confidence Interval; *mRS* modified Rankin scale; *BI* Barthel Index; *CES-D* Center for Epidemiologic Studies Depression scale; *MoCA* Montréal Cognitive Assessment; *TMT-A* Trail Making Test A; *TMT-B* Trail Making Test-B; *PSQI* Pittsburgh sleep quality index

**Table S10** | Influence of neuronal autoantibodies (all or NMDAR only) on outcome measures at 3 months post-stroke

|  | **Original Model** | | | **Imputed Model** | | | |
| --- | --- | --- | --- | --- | --- | --- | --- |
|  | **ß** | **95% CI** | ***p*-value** | **ß** | **95% CI** | ***p*-value** | **adjusted *p*-value** |
| mRS _All_ | 0.09 | -0.24 – 0.41 | 0.601 | 0.05 | -0.27 – 0.37 | 0.747 | 0.929 |
| mRS _NMDAR_ | -0.13 | -0.53 – 0.26 | 0.510 | -0.16 | -0.55 – 0.23 | 0.422 | 0.929 |
| BI _All_ | -1.49 | -8.20 – 5.21 | 0.660 | -1.59 | -8.39 – 5.21 | 0.644 | 0.929 |
| BI _NMDAR_ | 5.68 | -2.59 – 13.94 | 0.177 | 5.87 | -2.10 – 13.84 | 0.147 | 0.929 |
| CES-D _All_ | 1.56 | -2.27 – 5.40 | 0.422 | 2.34 | -1.35 – 6.02 | 0.212 | 0.929 |
| CES-D _NMDAR_ | 0.02 | -4.58 – 4.63 | 0.993 | -0.85 | -5.22 – 3.51 | 0.700 | 0.929 |
| MoCA _All_ | -0.06 | -1.31 – 1.20 | 0.927 | 0.12 | -1.23 – 1.48 | 0.856 | 0.950 |
| MoCA _NMDAR_ | 0.16 | -1.40 – 1.71 | 0.840 | 0.20 | -1.45 – 1.84 | 0.813 | 0.929 |
| TMT-A _All_ | 13.63 | -5.91 – 33.17 | 0.170 | 10.43 | -8.57 – 29.43 | 0.280 | 0.929 |
| TMT-A _NMDAR_ | -2.74 | -27.37 – 21.90 | 0.826 | -4.84 | -27.95 – 18.26 | 0.679 | 0.929 |
| TMT-B _All_ | -8.96 | -32.12 – 14.20 | 0.446 | -12.55 | -38.40 – 13.30 | 0.338 | 0.929 |
| TMT-B _NMDAR_ | -12.06 | -41.44 – 17.32 | 0.419 | -17.23 | -49.76 – 15.31 | 0.296 | 0.929 |
| PSQI _All_ | 0.13 | -1.14 – 1.39 | 0.842 | 0.06 | -1.23 – 1.35 | 0.927 | 0.955 |
| PSQI _NMDAR_ | 0.08 | -1.49 – 1.66 | 0.918 | -0.04 | -1.60 – 1.53 | 0.962 | 0.962 |
| EQ-5D-5L mobility _All_ | 0.14 | -0.32 – 0.59 | 0.550 | 0.08 | -0.35 – 0.50 | 0.713 | 0.929 |
| EQ-5D-5L mobility _NMDAR_ | 0.23 | -0.34 – 0.80 | 0.421 | 0.11 | -0.43 – 0.65 | 0.685 | 0.929 |
| EQ-5D-5L self-care _All_ | 0.10 | -0.34 – 0.53 | 0.667 | 0.07 | -0.35 – 0.49 | 0.742 | 0.929 |
| EQ-5D-5L self-care _NMDAR_ | -0.21 | -0.77 – 0.34 | 0.450 | -0.23 | -0.75 – 0.29 | 0.386 | 0.929 |
| EQ-5D-5L activities _All_ | 0.26 | -0.18 – 0.69 | 0.244 | 0.23 | -0.19 – 0.64 | 0.284 | 0.929 |
| EQ-5D-5L activities _NMDAR_ | 0.21 | -0.34 – 0.75 | 0.460 | 0.15 | -0.36 – 0.67 | 0.554 | 0.929 |
| EQ-5D-5L pain _All_ | 0.24 | -0.21 – 0.68 | 0.295 | 0.26 | -0.15 – 0.67 | 0.212 | 0.929 |
| EQ-5D-5L pain _NMDAR_ | 0.34 | -0.22 – 0.91 | 0.229 | 0.34 | -0.19 – 0.86 | 0.206 | 0.929 |
| EQ-5D-5L anxiety _All_ | 0.12 | -0.21 – 0.46 | 0.472 | 0.12 | -0.24 – 0.48 | 0.505 | 0.929 |
| EQ-5D-5L anxiety _NMDAR_ | 0.18 | -0.25 – 0.60 | 0.406 | 0.17 | -0.28 – 0.63 | 0.456 | 0.929 |
| EQ-5D-5L today_All_ | -2.06 | -10.28 – 6.15 | 0.620 | -0.49 | -8.08 – 7.10 | 0.898 | 0.955 |
| EQ-5D-5L today _NMDAR_ | 2.75 | -7.66 – 13.15 | 0.602 | 4.69 | -4.95 – 14.34 | 0.337 | 0.929 |
| RWT _ALL_ | 1.10 | -2.48 – 4.67 | 0.545 | 1.62 | -2.07 – 5.32 | 0.386 | 0.929 |
| RWT _NMDAR_ | 0.96 | -3.52 – 5.43 | 0.673 | 1.32 | -3.32 – 5.95 | 0.574 | 0.929 |
| Max. walking speed _ALL_ | -0.07 | -0.25 – 0.12 | 0.461 | -0.07 | -0.24 – 0.11 | 0.472 | 0.929 |
| Max. walking speed _NMDAR_ | -0.07 | -0.31 – 0.16 | 0.545 | -0.06 | -0.28 – 0.16 | 0.585 | 0.929 |
| Max. O2 uptake _ALL_ | -0.18 | -2.03 – 1.67 | 0.847 | 0.25 | -1.38 – 1.88 | 0.760 | 0.929 |
| Max. O2 uptake _NMDAR_ | -0.85 | -3.12 – 1.42 | 0.459 | -0.26 | -2.28 – 1.76 | 0.799 | 0.929 |
| FAC _ALL_ | -0.24 | -0.85 – 0.36 | 0.428 | -0.23 | -0.79 – 0.34 | 0.429 | 0.929 |
| FAC _NMDAR_ | 0.44 | -0.33 – 1.21 | 0.263 | 0.41 | -0.29 – 1.12 | 0.248 | 0.929 |
| 6 min walk distance _ALL_ | -5.60 | -44.28 – 33.09 | 0.775 | -2.88 | -37.79 – 32.04 | 0.871 | 0.950 |
| 6 min walk distance _NMDAR_ | -16.25 | -63.75 – 31.24 | 0.499 | -8.00 | -51.03 – 35.03 | 0.714 | 0.929 |
| Rivermead _ALL_ | 0.36 | -0.73 – 1.45 | 0.515 | 0.39 | -0.65 – 1.43 | 0.460 | 0.929 |
| Rivermead _NMDAR_ | 1.01 | -0.35 – 2.38 | 0.144 | 0.95 | -0.32 – 2.22 | 0.141 | 0.929 |
| Step count _ALL_ | -914.39 | -2744.1 – 915.3 | 0.324 | -807.60 | -2371.56 – 756.37 | 0.309 | 0.929 |
| Step count _NMDAR_ | -2047.74 | -4549.2 – 453.7 | 0.108 | -1485.60 | -3352.19 – 380.99 | 0.118 | 0.929 |
| REPAS _ALL_ | -1.28 | -3.77 – 1.21 | 0.312 | -1.03 | -3.39 – 1.34 | 0.391 | 0.929 |
| REPAS _NMDAR_ | -1.83 | -5.06 – 1.39 | 0.263 | -1.31 | -4.29 – 1.67 | 0.385 | 0.929 |
| Box and Block _ALL_ | 1.09 | -3.58 – 5.76 | 0.646 | 0.61 | -3.80 – 5.02 | 0.785 | 0.929 |
| Box and Block _NMDAR_ | 3.22 | -2.62 – 9.05 | 0.278 | 2.52 | -3.06 – 8.10 | 0.374 | 0.929 |
| MRC _ALL_ | -0.17 | -1.71 – 1.36 | 0.825 | -0.06 | -1.62 – 1.49 | 0.935 | 0.955 |
| MRC _NMDAR_ | 0.54 | -1.39 – 2.47 | 0.581 | 0.53 | -1.37 – 2.42 | 0.585 | 0.929 |
| Gait energy cost _ALL_ | 0.03 | -0.16 – 0.23 | 0.733 | -0.05 | -0.23 – 0.12 | 0.566 | 0.929 |
| Gait energy cost _NMDAR_ | 0.08 | -0.15 – 0.30 | 0.496 | -0.02 | -0.23 – 0.18 | 0.807 | 0.929 |

*95% CI* 95% Confidence Interval; *mRS* modified Rankin scale; *BI* Barthel Index; *CES-D* Center for Epidemiologic Studies Depression scale; *MoCA* Montréal Cognitive Assessment; *TMT-A* Trail Making Test A; *TMT-B* Trail Making Test-B; *PSQI* Pittsburgh sleep quality index; *RWT* Regensburg word fluency test*; FAC* functional ambulation category; *REPAS* resistance to passive movement scale sum score; *MRC* medical research council scale for muscle strength, sum score over 6 items

**Table S11** | Influence of neuronal autoantibodies (all or NMDAR only) on outcome measures at 6 months post-stroke in subpopulation of patients with haemorrhagic stroke (n = 19)

|  | **Original Model – patients with hemorrhagic stroke (*n* = 19)** | | | **Original Model – patients with ischemic stroke (*n* = 180)** | | |  |
| --- | --- | --- | --- | --- | --- | --- | --- |
|  | **ß** | **95% CI** | ***p*-value** | **ß** | **95% CI** | ***p*-value** | |
| mRS _All_ | 0.65 | -0.72 – 2.03 | 0.306 | 0.22 | -0.21 – 0.65 | 0.313 | |
| mRS _NMDAR_ | 1.11 | -1.09 – 3.31 | 0.277 | 0.07 | -0.44 – 0.59 | 0.776 | |
| BI _All_ | -6.81 | -49.31 – 35.68 | 0.716 | -2.76 | -9.44 – 3.91 | 0.413 | |
| BI _NMDAR_ | -33.39 | -96.51 – 29.73 | 0.251 | 0.97 | -7.47 – 9.40 | 0.821 | |
| CES-D _All_ | 6.35 | -21.11 – 33.81 | 0.556 | 4.68 | 0.77 – 8.60 | **0.019** | |
| CES-D _NMDAR_ | 17.10 | -14.35 – 48.56 | 0.206 | 4.81 | -0.07 – 9.69 | 0.053 | |
| MoCA _All_ | 0.83 | -1.25 – 2.92 | 0.351 | 0.67 | -0.85 – 2.20 | 0.385 | |
| MoCA _NMDAR_ | 1.61 | -1.53 – 4.74 | 0.245 | 1.39 | -0.48 – 3.26 | 0.143 | |
| TMT-A _All_ | 22.47 | -74.88 – 119.82 | 0.579 | 18.62 | -4.18 – 41.42 | 0.109 | |
| TMT-A _NMDAR_ | 37.98 | -108.06 – 184.03 | 0.533 | -15.23 | -44.21 – 13.74 | 0.300 | |
| TMT-B _All_ | 28.49 | -126.77 – 183.75 | 0.657 | 0 | -23.44 – 23.43 | 1.000 | |
| TMT-B _NMDAR_ | 21.84 | -231.70 – 275.38 | 0.834 | -6.81 | -36.92 – 23.29 | 0.655 | |
| PSQI _All_ | 0.01 | -2.15 – 2.16 | 0.994 | 1.55 | 0.13 – 2.97 | **0.032** | |
| PSQI _NMDAR_ | -1.10 | -4.28 – 2.08 | 0.390 | 1.94 | 0.16 – 3.72 | **0.033** | |
| EQ-5D-5L mobility _All_ | 1.86 | -0.36 – 4.08 | 0.088 | 0.48 | -0.05 – 1.00 | 0.074 | |
| EQ-5D-5L mobility _NMDAR_ | 2.55 | -1.13 – 6.23 | 0.145 | 1.10 | 0.46 – 1.74 | **0.001** | |
| EQ-5D-5L self-care _All_ | -0.07 | -1.82 – 1.68 | 0.962 | 0.18 | -0.28 – 0.65 | 0.436 | |
| EQ-5D-5L self-care _NMDAR_ | 0.54 | -2.24 – 3.31 | 0.661 | 0.24 | -0.36 – 0.83 | 0.428 | |
| EQ-5D-5L activities _All_ | 0.44 | -2.57 – 3.44 | 0.740 | 0.37 | -0.12 – 0.86 | 0.134 | |
| EQ-5D-5L activities _NMDAR_ | 1.78 | -2.58 – 6.14 | 0.367 | 0.61 | -0.01 – 1.23 | 0.054 | |
| EQ-5D-5L pain _All_ | 1.58 | -0.74 – 3.89 | 0.151 | 0.23 | -0.26 – 0.72 | 0.356 | |
| EQ-5D-5L pain _NMDAR_ | 0.70 | -3.77 – 5.18 | 0.722 | 0.75 | 0.13 – 1.36 | **0.018** | |
| EQ-5D-5L anxiety _All_ | -0.38 | -1.20 – 0.44 | 0.308 | 0.38 | 0.01 – 0.75 | **0.044** | |
| EQ-5D-5L anxiety _NMDAR_ | -0.30 | -1.71 – 1.11 | 0.629 | 0.30 | -0.18 – 0.77 | 0.222 | |
| EQ-5D-5L today_All_ | -18.93 | -45.52 – 7.66 | 0.136 | -7.72 | -16.54 – 1.10 | 0.086 | |
| EQ-5D-5L today _NMDAR_ | -23.34 | -74.67 – 28.00 | 0.318 | -12.38 | -23.61 – -1.16 | **0.031** | |
| Max. walking speed _ALL_ | -0.45 | -1.68 – 0.78 | 0.408 | -0.22 | -0.50 – 0.05 | 0.105 | |
| Max. walking speed _NMDAR_ | -0.87 | -2.27 – 0.53 | 0.179 | -0.33 | -0.66 – 0.01 | 0.054 | |
| Max. O2 uptake _ALL_ | -0.62 | -22.44 – 21.21 | 0.934 | 0.27 | -1.64 – 2.19 | 0.777 | |
| Max. O2 uptake _NMDAR_ | -1.21 | -22.85 – 20.44 | 0.871 | -0.26 | -2.72 – 2.20 | 0.834 | |
| 6 min walk distance _ALL_ | -324.09 | -940.66 – 292.49 | 0.218 | -7.93 | -60.47 – 44.61 | 0.765 | |
| 6 min walk distance _NMDAR_ | -231.4 | -1011.47 – 548.68 | 0.456 | -17.27 | -84.35 – 49.81 | 0.611 | |
| Rivermead _ALL_ | 1.51 | -2.96 – 5.99 | 0.450 | -0.10 | -1.43 – 1.22 | 0.877 | |
| Rivermead _NMDAR_ | 3.73 | -3.16 – 10.61 | 0.241 | 0.12 | -1.56 – 1.79 | 0.890 | |
| Box and Block _ALL_ | 3.10 | -17.79 – 23.99 | 0.736 | 0.21 | -6.54 – 6.96 | 0.952 | |
| Box and Block _NMDAR_ | -5.80 | -39.47 – 27.88 | 0.696 | 2.98 | -5.52 – 11.48 | 0.489 | |
| MRC _ALL_ | -3.55 | -11.30 – 4.20 | 0.315 | 0.10 | -1.75 – 1.96 | 0.911 | |
| MRC _NMDAR_ | -5.25 | -16.85 – 6.34 | 0.320 | 0.91 | -1.42 – 3.24 | 0.442 | |

*95% CI* 95% Confidence Interval; *mRS* modified Rankin scale; *BI* Barthel Index; *CES-D* Center for Epidemiologic Studies Depression scale; *MoCA* Montréal Cognitive Assessment; *TMT-A* Trail Making Test A; *TMT-B* Trail Making Test-B; *PSQI* Pittsburgh sleep quality index; *MRC* medical research council scale for muscle strength, sum score over 6 items
